# Can Polyhydroxyurethane-Derived Covalent Adaptable Networks Provide Environmental Benefits in Composite Applications?

**DOI:** 10.1021/acssuschemeng.5c01260

**Published:** 2025-07-15

**Authors:** Guillem Seychal, Pauline Bron, Olivier Talon, Nora Aramburu, Jean-Marie Raquez

**Affiliations:** † Laboratory of Polymeric and Composite Materials, Center of Innovation and Research in Materials and Polymers (CIRMAP), 54521 University of Mons, Place du Parc 23, 7000 Mons, Belgium; ‡ POLYMAT and Department of Advanced Polymers and Materials: Physics, Chemistry, and Technology, Faculty of Chemistry, 16402 University of the Basque Country UPV/EHU, Paseo Manuel de Lardizabal 3, 20018 Donostia-San Sebastián, Spain; § Materia Nova, Avenue Copernic 3, 7000 Mons, Belgium; ∥ WEL Research Institute, 1300 Wavre, Belgium

**Keywords:** non-isocyanate polyurethanes, composites, covalent adaptable networks, natural fibers, carbon fibers, depolymerization, life cycle assessment

## Abstract

Covalent adaptable networks (CANs) and CO_2_-derived polyhydroxyurethanes (PHUs) are often deemed as sustainable alternatives to conventional thermosets, particularly for composites made with epoxy (EP) matrices. However, the sustainability of CAN-based composites has never been assessed, nor has that of thermoset PHUs. Herein, we perform a life cycle assessment of PHUs, synergetic hybrid EP-PHU CANs, and EP in composite applications with either carbon or natural fibers (NFs) in order to address their syntheses, processes, and recycling. We demonstrate that producing cyclic carbonate monomers from epoxy and supercritical CO_2_ could be advantageous. PHUs provide potential environmental benefits to epoxy, but they are significantly limited by the energy inputs required for curing. Inversely, synergetic EP-PHU demonstrates noticeable environmental gain compared to EP and PHU-based composites and offers ideal recycling pathways. The chemical recovery of carbon fibers by oxidative depolymerization shows substantial benefits compared with virgin material production. When using NFs, mechanical recycling of CAN-based matrices is more suited due to the impacts of chemical recycling compared to virgin NF production, highlighting that the viability of a strategy strongly depends on raw materials and cannot be generalized easily. Strategies to further enhance the sustainability of composites are also proposed and discussed.

## Graphical abstract

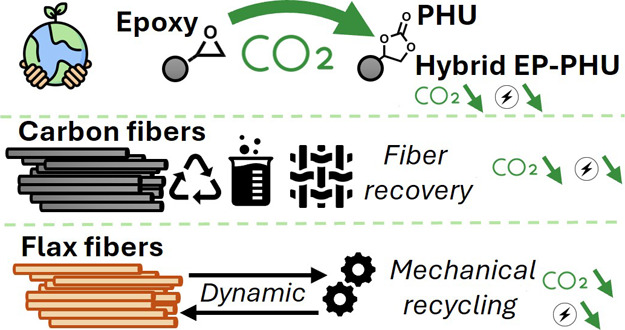

## Introduction

Human activities are leading to dramatic changes in the Earth’s climate and equilibrium.^1^ The need to drastically reduce the human footprint imposes developing greener materials.^1^ While fiber-reinforced polymers (FRPs) offer numerous benefits to improve the performances of structures and reduce their weight^2^ by promoting energy savings during the use phase, the current FRPs cannot be deemed sustainable.^3^ Glass and carbon fibers contain high embodied energy and account for significant global warming potential (GWP) during their production,^4^ while (thermoset) matrices severely limit the possible end-of-life (EoL) scenarios.^5^

Over the last decades, new polymers and chemistries have emerged, ushering new opportunities in materials, particularly in composites.^6^ Using renewable feedstocks such as natural fibers (NFs) for reinforcements^7^ or bioalternatives to petrochemicals for the matrices,^8^ significant environmental benefits have been demonstrated in various laboratories and occasionally scaled to industrial applications.^9^ NFs are, in most cases, significantly greener than synthetic glass fibers.^10^ Moreover, these NFs can be combined with (biobased) thermoplastics to obtain recyclable^11,12^ and eventually biodegradable materials,^13^ but the resulting NF composites usually do not show satisfying properties.^11^ By contrast, biobased thermosets, such as epoxides (EP), ideally derived from plants, provide much higher stability and mechanical properties^14^ but are inherently not recyclable.^15^ In this regard, increasing interest in covalent adaptable networks (CANs) that reassemble the superior properties of thermosets with the advanced recyclability features of thermoplastics^16^ opens new opportunities for the management of decommissioned composite structures.^17^ Developing recycling strategies such as pyrolysis or solvolysis also enables foreseeing recirculation opportunities for the high-added-value fibers, particularly in carbon fibers.^18^ Recovery of carbon fibers through pyrolysis or solvolysis processes has been shown to produce fewer emissions than virgin carbon fiber production and appears as a viable strategy to mitigate the environmental burden of carbon fiber-reinforced polymer (CFRP) structures.^19,20^ However, because NFs are more sensitive to high temperatures, pyrolysis and solvolysis are inapplicable to NFs.^21^

Our research team has demonstrated that polyhydroxyurethanes (PHUs) could provide a fortunate platform for a new generation of FRPs.^22^ The hydroxyurethane moieties in the backbone enable high adhesion at the fiber/matrix interface, as well as matrix-adaptable behavior that allows the composite to be reshaped and welded afterward. PHUs have been shown to be valuable alternatives to EP resins. The literature generally considers the production of cyclic carbonates, key building blocks for PHU, to be green, as they are easily produced from the cycloaddition of CO_2_ in EP monomers.^23,24^ This can be even performed quantitatively under solvent-free conditions using supercritical CO_2_.^25^

The additional steps to transform EP into cyclic carbonates require energy to bring CO_2_ to its supercritical state and should be considered. Additionally, the polymerization protocol to obtain PHUs requires a higher temperature and a longer time than epoxy.^26^ As PHUs have not been shown to be industrially relevant for the composite manufacturing industry, a hybridization strategy has been developed.^27^ This hybridization, consisting of a homogeneous PHU/EP copolymer, reduced the viscosity to a range suitable for resin transfer molding and significantly simplified the curing protocol.^28^ Moreover, the dynamicity of the networks was enhanced, allowing faster and more efficient thermo-mechanical reshaping. Finally, we demonstrated that the networks were cleavable under mild conditions and that flax and carbon fibers could be retrieved with minor degradation.

Nonetheless, this claimed sustainability has never been assessed quantitatively, and life cycle assessment (LCA) on sustainable materials has remained overlooked. Renewable sourcing of raw materials does not systematically lead to environmental benefits, while the sustainability of material recycling lies in the difficult balance between the impact of the recycling process and the savings from the recycled products. Despite the claimed sustainable potential of CANs, only Vora et al.^29^ investigated the potential of polydiketonamine dynamic networks to compete with commodity polymers. At the same time, chemical recycling has been overlooked in quantitative sustainability assessment. La Rosa et al.^30^ demonstrated that recycling fibers from composite cured with the Recyclamine could be favorable. Still, the synthesis of the Recyclamine hardener was not modeled, neglecting the impact of chemical synthesis. PHU thermoplastic displayed some potential to compete with conventional PUs^31^ but is not yet competitive property-wise. Investigating all aspects of CAN-based composites from sourcing to recycling seems in that sense a necessity to properly advance the sustainable field.

However, all those strategies for composites and their subsequent environmental footprint largely depend on the choice of raw materials, manufacturing steps, service life, and finally, the management and valorization of decommissioned parts.^32^ Therefore, the question arising from these recent developments concerns the actual environmental footprints of CANs, PHUs, and hybrid EP-PHUs compared to epoxy in composite applications for both natural and carbon fibers. Furthermore, carbon and natural fibers are suitable for different applications and possess significantly different environmental burdens. Hence, one must determine whether strategies to lower the environmental footprint of one composite can be extrapolated to another. An LCA is performed herein to provide a simplified audit of the production and recycling of PHU-based CANs and composites. The work also aims to discuss future needs and efforts in the field of sustainable CAN composites to reduce environmental impacts (EI). An overview of materials choices and EoL strategies is displayed in Figure 1.

**Figure 1:**
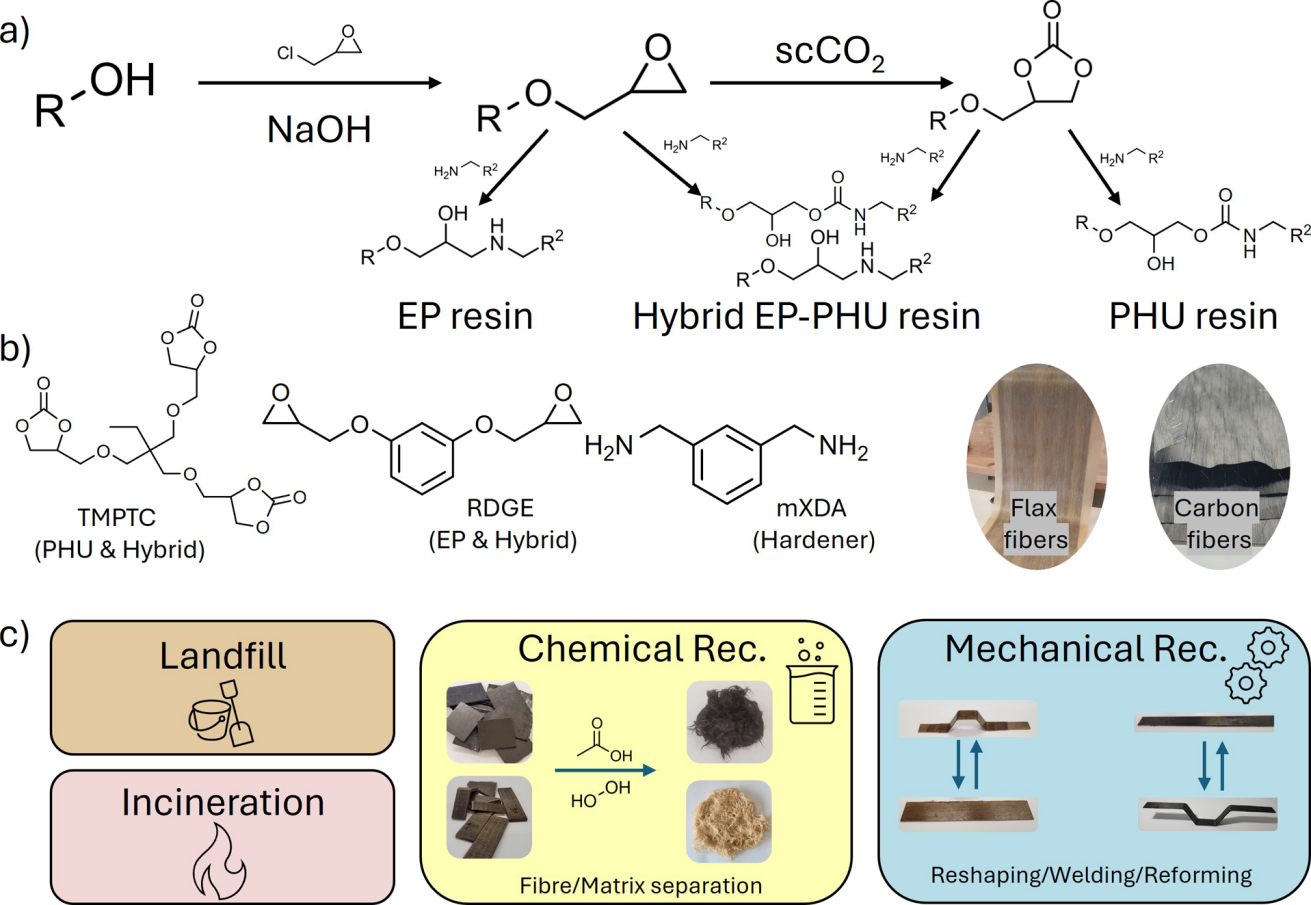
Overview of the modeled materials and steps. (a) Chemical steps for epoxy and cyclic carbonate synthesis, (b) constituents of the materials considered, and (c) overview of the proposed end-of-life scenarios.

## Methods and Scopes

### Objectives and Scope Definition

This environmental evaluation encompasses the production of the building blocks of the matrices, the production of the fibers, the curing protocol, and end-of-life scenarios for the composites. Contribution analyses were performed to identify the most impacting materials and process steps. Comparisons are made for various EoL scenarios, different matrices (EP vs PHU/EP hybrid), and both flax and carbon fibers. As such, the ranges of applications for flax and carbon fiber composites may differ. Therefore, comparing these two types of composites was considered to be out of scope.

### System Boundaries

The LCA encompasses the different stages of the composite value chain to better understand the influence of chemistry and design choices. The first part focuses on understanding the environmental effect of a simple drop-in change from epoxy to PHU or hybrid EP-PHU and the major contributors to each material. As such, a cradle-to-gate approach was chosen. Second, the full life cycle (excluding the use phase) was modeled through a cradle-to-grave approach on the hybrid EP-PHU-based composite to discuss the impacts of EoL managements.

Our previous work^22,27^ demonstrated that hybrid EP-PHU leads to a slight property improvement compared to EP. To simplify the modeling, the properties of the resins and composites were hypothesized as being equal. Therefore, there is no change in the design of the functional units (taken as 1 kg composite plates of similar stiffness). The use phase is assumed to be identical and out of the scope of our work. Equally, transportation and specific manufacturing plants are not considered. To compare the recycling process, which leads to long (>5 cm) nonoriented fibers, a quality factor of 0.7 was used, that is, 1.0 kg of the composite using recycled fibers performs like 0.7 kg virgin fiber composite. Moreover, other production steps, such as weaving or sizing of the fibers, are neglected.

Equally, the model represents the current development stage, that is, at the lab scale. The results might significantly differ in larger-scale facilities. Because the technology has been developed in Europe, data were chosen to fit the European market and might vary in other localizations. Figure 2 shows the steps included in the system boundaries for the LCA study. The sequestrated carbon in flax and cyclic carbonate was not considered.

**Figure 2:**
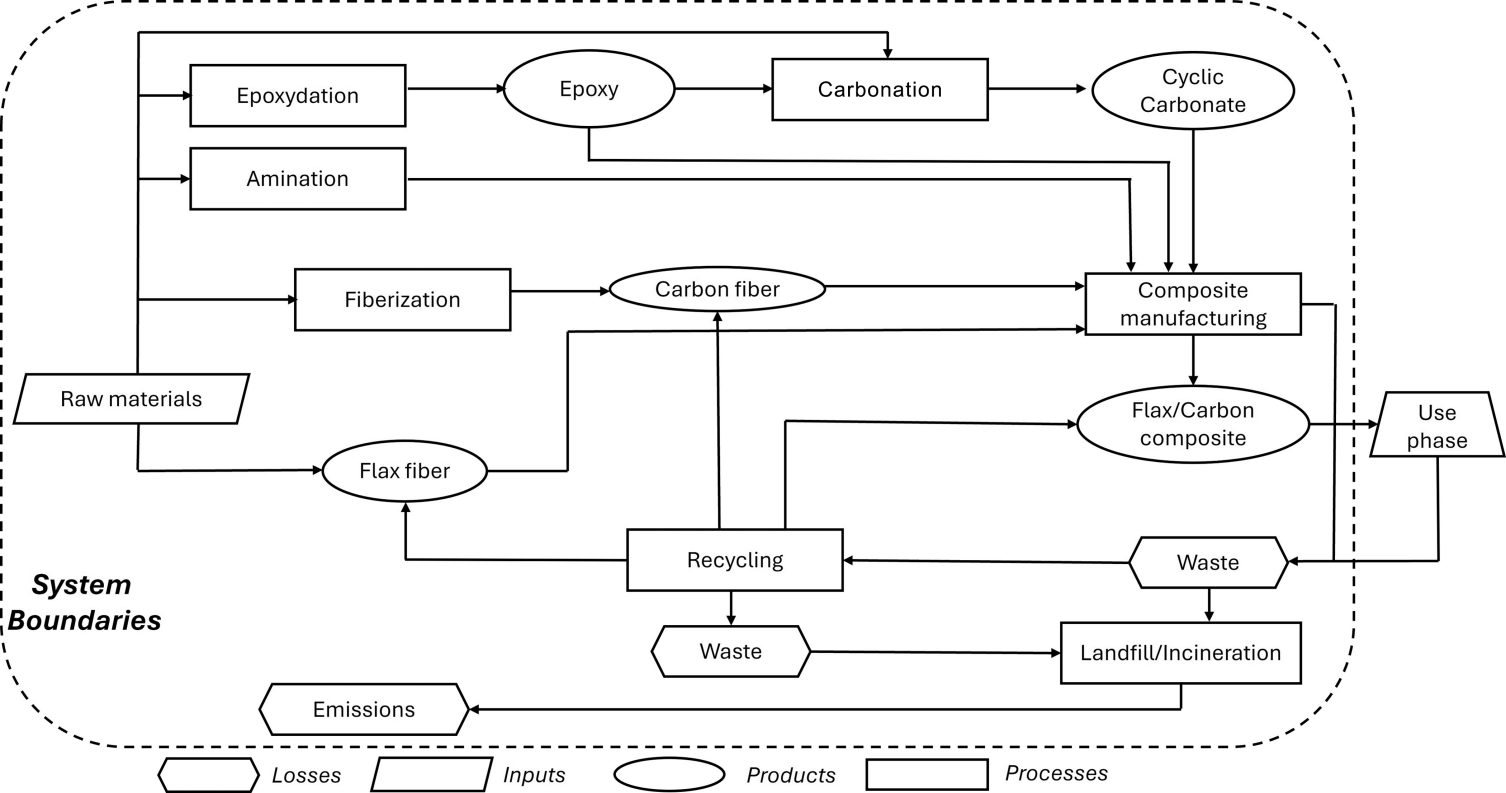
Boundaries of the studied system and schematic representation of the overall processes and life cycle.

### Scenarios

The scenarios consist of three main phases. The first phase is the raw material production phase, which encompasses all of the raw materials that are used in the composite and their preparation. The second phase is the composite manufacturing phase, which involves the energy consumed during the production process, as well as any energy losses incurred. The final phase is the disposal phase, which includes the energy and materials required for recycling or waste treatment of the composite. Carbon and flax fibers are studied separately. For the two first phases, a preliminary cradle-to-gate analysis is performed to compare the EI of PHU, EP, and hybrids and the impact of the production steps. The carbonation of epoxy is considered. Neat resins are also compared. For composites, flax-reinforced polymers are evaluated with EP, PHU, and hybrid EP-PHU matrices. Only EP and EP-PHU are deemed as carbon fibers.

To investigate the different EoL strategies and the scenarios as a whole, a cradle-to-grave LCA is performed on the hybrid EP-PHU with flax and carbon fibers. The landfill of EP-based composites is taken as a reference scenario. Four different EoL scenarios were considered:

- Landfill: For each case (flax or carbon fibers), two virgin composite materials are considered simultaneously, one of 1 kg and one of 0.7 kg. Decommissioned materials are considered to be landfilled as inert waste in the European market.
- Incineration (flax composite only): Similarly, two virgin composite materials are considered simultaneously. Decommissioned materials are considered to be incinerated with energy recovery in the European market.
- Thermo-mechanical: A virgin composite material (1 kg) for both fibers is considered and later transformed through a thermo-mechanical process into a new generation with a 0.7 quality factor. Finally, the second material is decommissioned into inert waste in the European market.
- Acidolysis: A virgin composite material (1 kg) is considered. Oxidative depolymerization is employed to retrieve the fibers (flax or carbon fibers). The fibers are reused to produce a new composite with a 0.7 quality factor. The second generation is then treated in the inert waste European market.

### Analysis Method and Environmental Data

The LCA methodology is structured according to the ISO standards (14040). The LCA study was performed using Simapro 9.6 software. Ecoinvent 3.10 was used as a background database in the Cut-Off version as provided with Simapro. When unavailable in the Ecoinvent database, data were either collected from lab experiments (energy measurements, developed processes) or the literature. Life Cycle Impacts were calculated with the Environmental Footprint 3.1 assessment method in the version provided by Simapro.

Background data specific to the European market were primarily selected when available (RER); otherwise, global market data (GLO) were used. Electric energy was chosen as the European mixed market group. Data related to input materials were calculated at each step from the different protocols using the mass of raw materials to produce 1 kg of products. Energy-related data were directly measured using a wattmeter apparatus installed on the laboratory equipment with a 0.1 kWh resolution. The CO_2_ credit stored through photosynthesis and the carbonation process is not taken into account. The cutoff was chosen to zero the burden of recycled materials (i.e., only the recycling treatment imparts the EI of subsequent uses). For analyses, all 16 indicators listed in Table 1 were computed. However, to simplify the results and in light of the uncertainties to quantify such parameters from lab results, water use (WAT), human toxicity (HT-c & HT-nc), freshwater ecotoxicity (FWT), and land use (LU) are not graphically represented. All data are reported in the corresponding tables.

**Table 1:** Abbreviations of Environmental Impact Indicators

acronym	name	unit
CC	Climate Change (GWP100)	kg CO_2_ eq
ODP	Ozone Depletion	kg CFC11 eq
PM	Particulate Matter	disease inc.
IR	Ionizing Radiation	kBq U-235 eq
POF	Photochemical Oxidation	kg NMVOC eq
AC	Acidification	mol H + eq
FE	Freshwater Eutrophication	kg P eq
ME	Marine Eutrophication	kg N eq
TE	Terrestrial Eutrophication	mol N eq
RES-f	Resources fossil	MJ
RES-m	Depletion of abiotic resources	kg Sb eq
WAT	Water Use	m3 depriv.
LU	Land Use	Pt
HT-nc	Human Toxicity noncarcinogenic	CTUh
HT-c	Human Toxicity carcinogenic	CTUh
FWT	Freshwater Ecotoxicity	CTUe

## Life Cycle Inventory

This section summarizes the input data exploited to model the scenarios. The inputs are summarized in the first section of the Supporting Information.

### Chemical and Precursor Synthesis

#### Epoxy

Epoxies were modeled similarly to diglycidyl ether of bisphenol A (DGEBA), which is already available in the Ecoinvent database. Trimethylolpropane (TMP) is not present in the database, and pentaerythritol was chosen as a proxy, both syntheses being closely related. Shortly, resorcinol or trimethylolpropane is reacted with epichlorohydrin (ECH). The reaction is commonly performed in a large excess of ECH. The excess is considered to be recycled (not modeled). Sodium hydroxide (2 equiv/epoxy group) is used to close the epoxy ring. Benzyltriethylammonium chloride (TEBAC) catalyzes the reaction between TMP and ECH but is not required for resorcinol.^33^ The energy requirements for the synthesis were assumed to be identical to the one used in the Ecoinvent model for DGEBA. Resorcinol diglycidyl ether (RDGE) has been modeled to be the epoxy, while trimethylolpropane triglycidyl ether (TMPTGE) is used as the precursor to cyclic carbonate.

#### Cyclic Carbonates

Cyclic carbonates are straightforwardly obtained from the cycloaddition of CO_2_ within epoxy.^25^ TMPTGE was used as the epoxy precursor to obtain trimethylolpropane tricarbonate (TMPTC). The typical synthesis is performed on the kilogram scale in a 2 L high-pressure stainless steel reactor. The epoxy is loaded with a catalyst, typically tetrabutyl ammonium iodide (here, TEBAC is used in the model). CO_2_ is then injected under pressure into the reactor while heating. The reactor is stabilized at around 80 °C and 100 bar to reach CO_2_ supercritical conditions. The reaction is typically performed between 15 and 24 h^34^ with quantitative yields. No solvent is required or purification steps.

#### Amine

m-xylylene diamine (mXDA) was used as the hardener for all resins. The synthesis of amines is usually performed from the hydrogenation of nitriles^35^ in the presence of a nickel catalyst. Nitriles are mainly produced via the SOHIO process. mXDA is produced from the ammoxidation of xylene, using ammonia and dioxygen to yield isophthalonitrile which is further hydrogenated to mXDA. The inputs were taken from the literature.^31^

#### Carbon Fibers

Carbon fibers are a high source of discrepancies in the literature, with several authors having reported drastically different results. The industrial secrecy around production also makes accurate estimation difficult. Recently, Jacquet et al.^4^ have proposed a justified and transparent inventory based on Ecoinvent background data sets. This inventory was selected as the reference. It includes acrylonitrile and vinyl acetate as the precursors to polyacrylonitrile (PAN) fibers, nitrogen, and steam to stabilize the production of fibers and electricity and heat required to carbonize PAN fibers into carbon fibers. The results from their work highlight a global warming potential of 72 kgCO_2_eq and a cumulative energy of 1176 MJ per kg of carbon fibers, which is at the upper range of commonly estimated impacts for CF.^36^ The modeling of carbon fiber aims to be representative of the order of magnitude and should not be taken for comparing carbon fiber composite EI outside of this work as it might not fully represent all impacts.^4^

### Polymers and Composite Manufacturing

All resins were cured in equimolar quantities of the monomer and hardener. The EP resin comprises RDGE and mXDA. RDGE is an aromatic epoxy that is often considered an alternative to DGEBA.^33,37^ The PHU contains TMPTC and mXDA. The hybrid EP-PHU incorporates RDGE and TMPTC in a 50/50 mass ratio, cured with mXDA. Flax composites are modeled with a fiber weight fraction of 60%, and a 70% mass fraction was used for CFRP composites. The curing is identical for neat resins and composites and is assumed to be performed in thermo-compression using a pressure of 4 bar. For the EP resin and the hybrid, the curing is performed for 30 min in the heating press at 80 °C followed by 1 h at 160 °C in an oven.^28^ The PHU was cured for 2 h at 80 °C, followed by 1 h at 100 °C and 1 h at 150 °C in an oven.^22^ The energy inputs were measured from our lab equipment. The energy inputs are considered to be the same for neat resins and composites and were measured to align with the requirements of composite manufacturing. Therefore, energy inputs for pure thermosets might be underestimated and should be taken with care. For composites, 10% production waste was accounted.

### Recycling Phase

Two recycling methods were considered: thermo-mechanical and chemical. The hybrid EP-PHU is a CAN. For the two methods, collecting, sorting, cleaning, and potential preliminary preparation steps are neglected.

Unlike EP thermosets, thermo-mechanical recycling can be considered to a certain extent for hybrid EP-PHU. Two main strategies could be regarded: the first one includes the shredding of the composite and the compression into a low-grade filled polymer, similar to a short fiber-reinforced thermoplastic. However, this strategy leads to a drastic downgrade in the material quality, which enables the consideration of these as-processed materials for reuse in only low-cost, low-performance applications. Moreover, the matrix weight fraction in the virgin structural composite is low, which tends to decrease the efficiency of such a method. Therefore, a repurposing approach was preferred. In that case, the laminate is collected and reshaped and welded into a new material. This approach is even more promising as it keeps the fiber length, and the thermo-compression step can consolidate the matrix, reducing porosities and cracks in the matrix generated during the first use phase.^38^ The process involves a unique step of thermo-compression at 180 °C for 30 min. A quality factor of 0.7 was applied to account for the potential decrease in the material properties. This quality factor means that it is assumed that 1 kg of recycled product would have a functional performance equivalent to 0.7 kg of virgin material. An additional 30 wt % loss accounted for cuts and other preliminary preparations and finishes.

As hybrid EP-PHU was more suitable for depolymerization under mild conditions, allowing the recovery of carbon or natural fibers, chemical depolymerization was also modeled. A depolymerization mixture of acetic acid (HAc) and hydrogen peroxide is prepared (80:20) and heated at 60 °C for 4 h. The network is cleaved, and the fibers can be recovered by filtration. The HAc is recovered (90% efficiency) and re-employed. The degraded polymer solution is treated as a hazardous solvent mixture (incinerated). A new composite is prepared using 60 wt % of fibers and virgin hybrid matrix. A conservative quality factor of 0.7 is applied.

## Results and Discussion

### Production of Epoxy and Cyclic Carbonates

The production of cyclic carbonates for PHU resins demands the carbonation of epoxy monomers.^24^ The synthesis only requires the use of CO_2_ under supercritical conditions to serve as both the solvent and the reactant. However, because of the CO_2_ thermodynamic stability, a catalyst and energy are required to form the desired product.^25^ Supercritical conditions are advantageous as they limit the generation of waste and purification steps.^23^ Replacing epoxy resin with PHU in composites requires this additional carbonation step, which must be first environmentally assessed.

The contribution of each constituent in the calculated impacts of TMPTC is represented in Figure 3b. The main contributor to the overall EI of TMPTC remains the epoxy monomer, accounting for 80% of the CC indicator and being a major contributor to all indicators. The use of CO_2_ as the reactant can be almost considered neutral, with a minor contribution to all indicators. The catalyst does not make a significant contribution either. While catalyst selection should be driven by cost, toxicity, and catalytic activity, mainly to shorten the reaction time, it does not appear to have a strong influence on the EI. The energy consumption related to the reaction accounts for 12% of the CC and 17% of the resource depletion indicators. The larger contribution to the overall EI of cyclic carbonates originates from the epoxy precursor.

**Figure 3:**
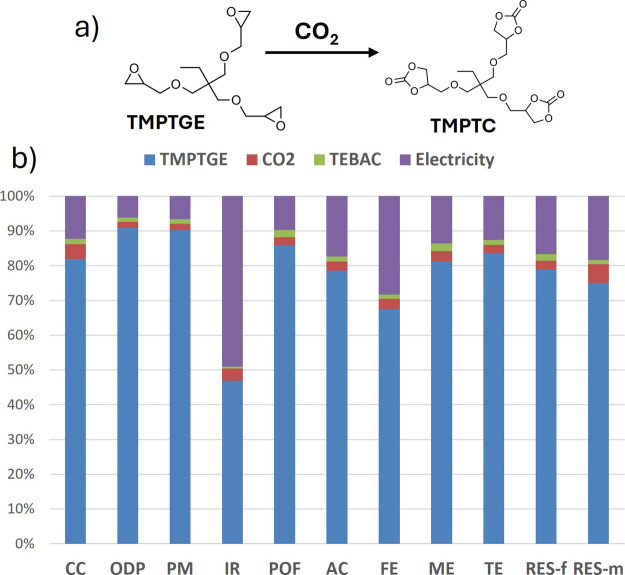
Environmental impacts of TMPTC production. (a) Carbonatation of TMPTGE into TMPTC, and (b) contribution of each input in TMPTC.

The production of cyclic carbonates results in a mass gain for the monomers due to the CO_2_ fixation. Here, 1.1 kg of epoxy yielded approximately 1.6 kg of cyclic carbonates. As the objective is to replace in a drop-in approach (i.e., direct replacement) the epoxy matrix with a PHU-based one, the epoxy monomer and the cyclic carbonates were compared and normalized to 1 kg of starting monomer. The results are presented in Figure S3 and summarized in Table S15. The values of the CC indicator and cumulative energy for TMPTGE are consistent with the literature for other epoxy monomers, which are commonly estimated to be 4–8 kgCO_2_eq/kg and 70–150 MJ/kg, respectively.^39,40^ Interestingly, the cyclic carbonate displays lower environmental impacts than its epoxy precursor owing to the efficient carbonation process and the mass increase from CO_2_ incorporation. In that sense, the results obtained in the present work demonstrate that the supercritical carbonation process itself does not add any detrimental environmental burden and can, to some extent, be regarded as a greener process.

Other pathways using low-pressure CO_2_ have been developed;^41^ however, they require the use of solvents such as ethyl acetate and a purification step, resulting in lower yields. For reference, the process was modeled (see Figure S4) and compared to the scCO_2_ method. Despite lower energy consumption, the strategy led to increased impacts for almost all indicators, especially for CC, with a 70% increase. Therefore, scCO_2_ currently appears to be the most promising strategy.

The results obtained from this first cradle-to-gate analysis demonstrate that the production of cyclic carbonates might be beneficial in mitigating the EI of thermosets. However, to consider this strategy truly sustainable, the toxicity of cyclic carbonates should be addressed. Cyclic carbonates are commonly regarded as fairly nonhazardous,^42^ with ethylene carbonate, a rather well-known chemical, considered safe. Yet, this should be confirmed for other carbonated monomers. Moreover, while being promising, these results need to be extended first to the material level, in accounting for the process and other constituents, and finally to the entire life cycle.

### Comparison of Epoxy, Polyhydroxyurethane, and Hybrid EP-PHU Resins

The literature^22,26,41,43^ documented the lengthy curing times at elevated temperatures of pure PHU thermosets and underlined them as a problem in the production of composites. Such issues were tackled through the synergetic hybridization strategy,^27^ which facilitated and accelerated the curing process. Yet, it is essential to evaluate the share of each resin’s inputs into the EI, including the energy required to cure them, which might hamper future benefits. The contribution analysis of the impacts of each cured resin and the compared impacts of the three systems is presented in Figure 4.

**Figure 4:**
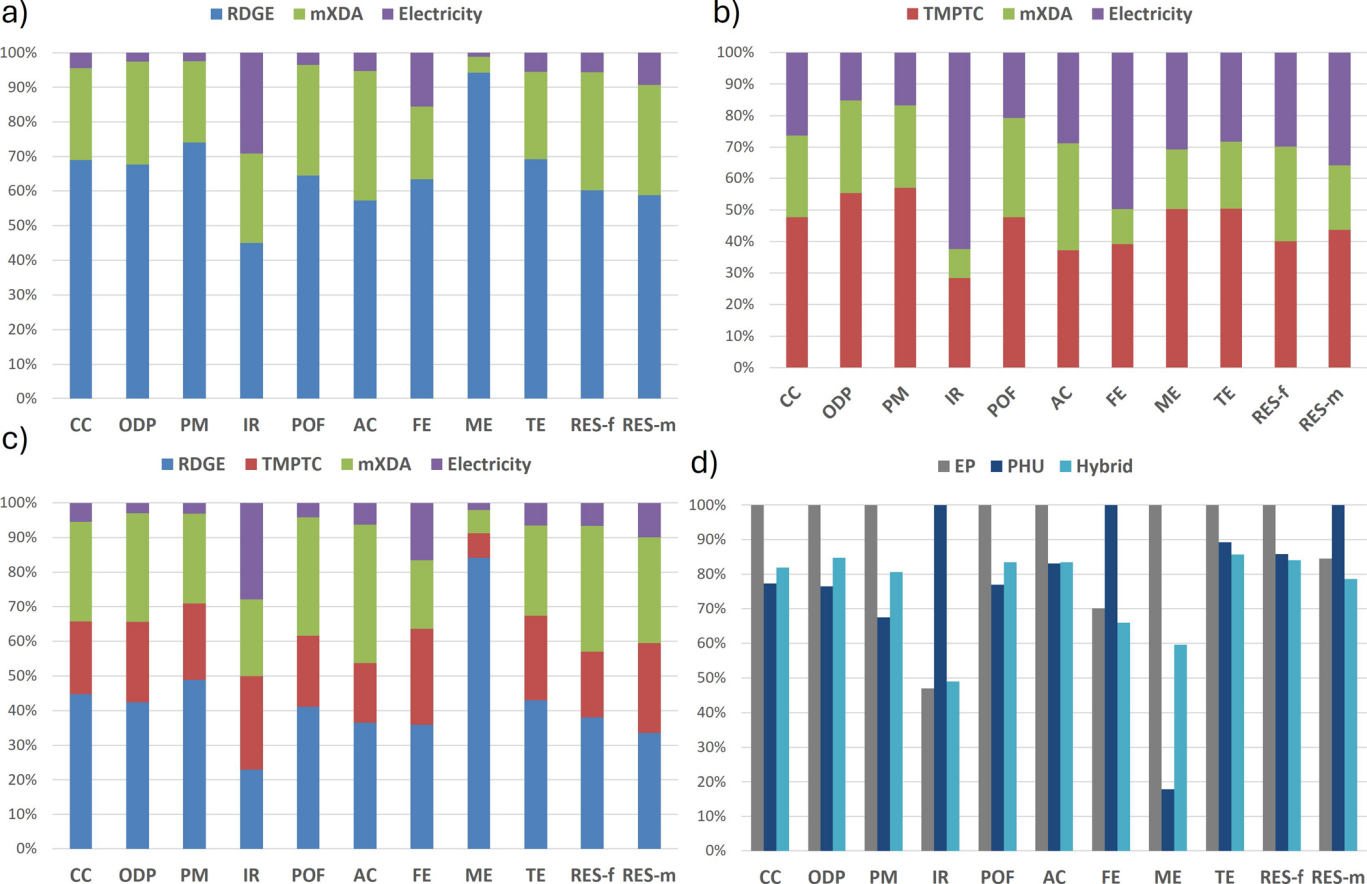
Contribution of each constituent of the cured resins on the overall EI of the resins. (a) Epoxy resin, (b) PHU resin, (c) hybrid EP-PHU, and (d) comparison of the impacts of the different resins (for 1 kg of resin).

About 10.4 kgCO_2_eq/kg of cured epoxy resin are estimated with 10 MJ/kg required, in the upper range of typical epoxy resin.^39,40^ The main contributor in EP is RDGE, representing 70% of the CC indicator and 60% of RES-f, and superior to 50% in all other categories. mXDA is the second contributor, representing about 25% of most indicators. The EI of the energy required to cure the epoxy is marginal, accounting for about 4% of emitted kgCO_2_eq This is sounded as the European energy mix is rather decarbonated, with about 67% coming from renewable energy or nuclear.^44^ Similarly, RDGE appears as the highest contributor to the hybrid resin, representing 40–50% of the EI despite making up about a third of the mass. The influence of mXDA and electricity is similar to that of EP with 27 and 5%, respectively. Interestingly, TMPTC represents less than 20% of the EI sources. In the pure PHU, the share of the curing energy substantially increases in all categories, becoming an important source of EI, up to 26% for CO_2_eq In that case, TMPTC represents 48% of the EI, significantly less than RDGE in the EP. The curing mXDA represents around 26% of the CC and consumed energy.

Both the hybrid and the PHU resin allowed a drastic reduction of most EI, ranging by 20 to 50% compared to the epoxy benchmark, apart from ionizing radiation, freshwater eutrophication, and abiotic resource depletion for the PHU. The CC is reduced by 28 and 21% for the PHU and the hybrid, respectively. The PHU exhibits slightly lower CC results than the hybrid but should be considered similar given the potential uncertainties in modeling.

In the hybrid, the presence of RDGE imparts a high EI, which could be expected to impede the environmental benefit of the resin. The use of TMPTC in parallel to improving the curing protocol compared to neat PHU enables compensation for the presence of RDGE and makes the hybrid competitive with the PHU. This is promising, as previous work has demonstrated that pure PHU might not be relevant at the current development stage, but the hybrid strategy can be implemented faster while keeping the environmental benefits. The results demonstrate that PHU and their hybrids are valuable approaches to reducing the environmental footprint of epoxy-based resins.

### Benefits of PHU Chemistry in NF Composites

NFs, particularly flax, have been widely demonstrated as a greener alternative to synthetic fibers.^10,45,46^ Particularly, flax fibers have low embodied energy and account for minimal CC effects during their life cycle, sometimes resulting in negative GWP due to carbon sequestration.^10^ Therefore, in a flax fiber composite, the matrix used is responsible for a major share^47^ in the majority of indicators (apart from some exceptions such as land use or water consumption) and should be optimized to ensure the full sustainability of the resulting composite.

EP, PHU, and hybrid matrices were evaluated with a scutched long fiber reinforcement in a cradle-to-gate approach. Results for individual laminates and the normalized comparison are shown in Figure 5. It is assumed that the PHU or the hybrid would be used as a direct replacement for the EP resin with no change in application. This assumption favors the benchmark resin, as the literature demonstrated improvements in the properties of the PHU and the hybrid.^22,27^ Moreover, it must be noted that the PHU resin is considered for reference purposes only, as it might not be applicable to real-world scenarios due to economic and technological reasons.

**Figure 5:**
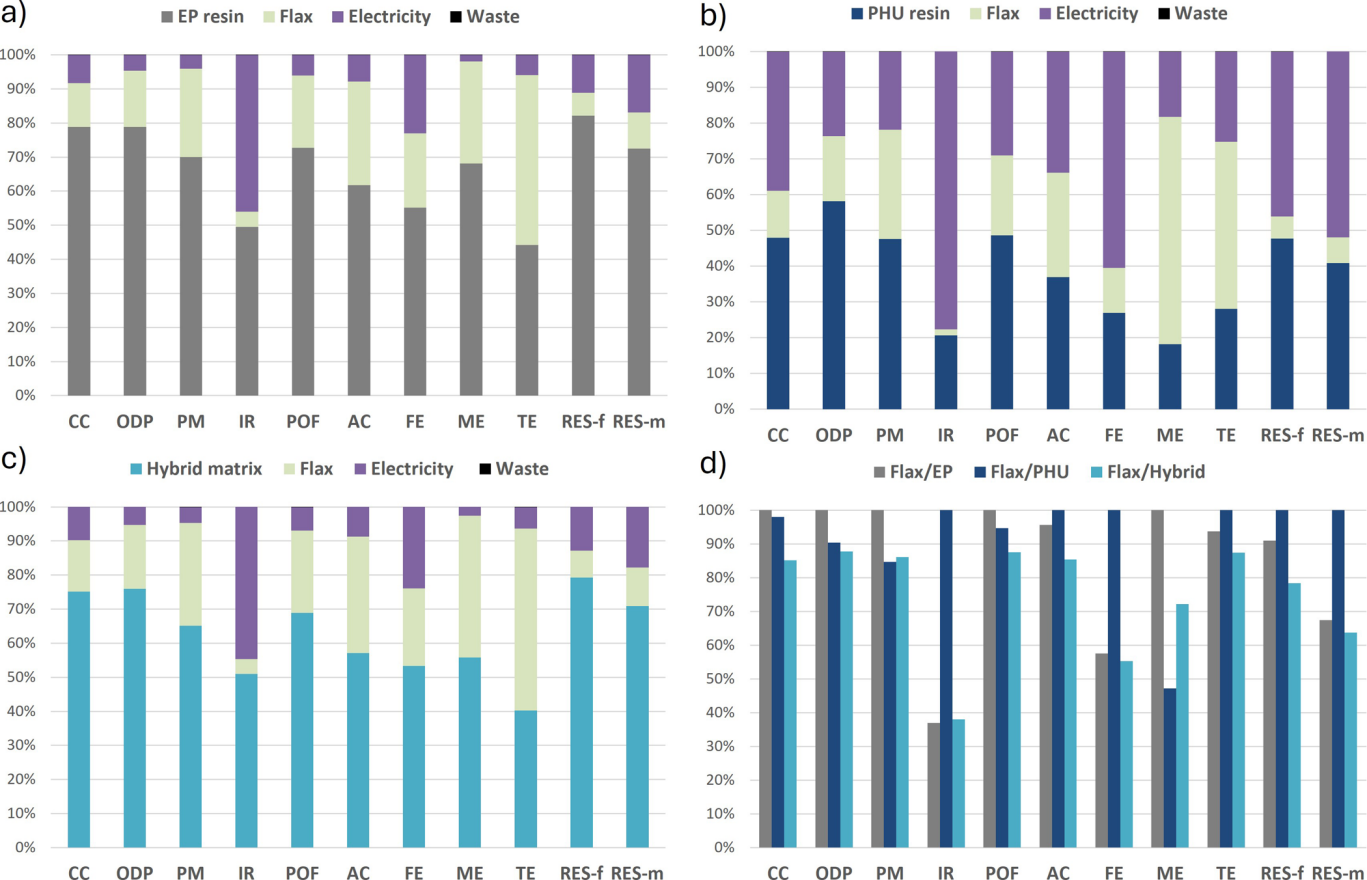
Distribution of each constituent of the flax-based composites on the overall EI. (a) Flax/epoxy composite, (b) Flax/PHU composite, (c) Flax/hybrid composite, and (d) comparison of the different flax composite.

For all composites, the flax fibers contribute about 12–15% of most indicators. As discussed, the resin is the major contributor, which highlights the need to focus on low EI resins for NFC. The EP and hybrid resins account for both roughly 75% of the CC indicator and 80% of the fossil resource indicator. For flax/PHU, the low EI of the PHU resin reduced its share by about 50%. It remains rather high considering that the resin constitutes only 40% of the mass in all composites. The PHU points out the detrimental effects of the more energy-intensive curing protocol. In PHU, the electricity required to cure the laminate accounts for 40% of the CC and is a significant contributor in all indicators.

When the impacts of the three systems are compared, using the hybrid resin demonstrates a substantial decrease in all EIs. Flax/EP showcases the highest CC indicator, photochemical oxidation, and marine eutrophication. Around 5.5 kgCO_2_eq/kq are estimated for the Flax/EP, with 96 MJ/kg. These values are in the upper range of the literature,^40,48^ but the comparison might not be entirely feasible due to the small scale used here to model the scenarios and the reliance on mostly in-house models that commonly have higher EI than database ones. Therefore, the comparison should remain internal to the materials in the present study.

The flax/PHU could have been expected to be the best performing material. However, the results are totally inhibited by the detrimental amount of energy for the curing step, and no significant difference can be observed between the calculated climate change indicator and the reference. The PHU-based laminate even demonstrates the worst results in terms of ionizing radiations, acidification, freshwater and terrestrial eutrophication, and fossil energy. The fossil resource depletion indicator is estimated to be 106 MJ/kg, which represents a 10% increase compared to flax/EP. These results showcase that using a low environmental footprint matrix might not lead to environmental benefits if the switch leads to a change in the process with higher energy consumption.

The hybrid, on the other hand, indicates more promising results. All indicators decrease compared to those in both PHU and EP matrices. The CC indicator and fossil resource depletion are reduced by 15% compared to the EP resin as well as eutrophication. About 4.7 kgCO_2_eq/kg are estimated to be emitted during the production of flax/PHU laminates. The results demonstrate that the synergetic hybridization that was developed herein has not only opened the door to obtain efficient and easily accessible CANs, with characteristics relevant to the composite industry, but also confirmed the initial hypothesis that PHU chemistry, with optimization, represents a sustainable pathway for NF composites.

### Benefits of Hybrid EP-PHU in Carbon Fiber Composites

CFRPs are known for their high EI, due to the significant contribution of PAN-based carbon fiber.^4^ It was demonstrated that upon careful design, the EI mainly issued from the raw material production and composite manufacturing could be compensated by energy savings during the use phase^49^ but remains not systematic.^50^

Our research group previously demonstrated that PHUs are irrelevant for CFRPs,^22^ whereas hybrids have prospects.^27^ In this regard, only the EP and hybrid resins were studied in this section. It must be noted that CFs, which represent 70% of the composite’s mass, account for 93% of the CC indicator and fossil resource depletion and are the major contributors to all indicators. The results for the CF/Hybrid reaction are shown in Figure 6a. Around 50 kgCO_2_eq/kg of the composite was estimated with a cumulative energy of around 900 MJ/kg. The results are in the upper range of different studies but remain within the same order of magnitude.^49,51^

**Figure 6:**
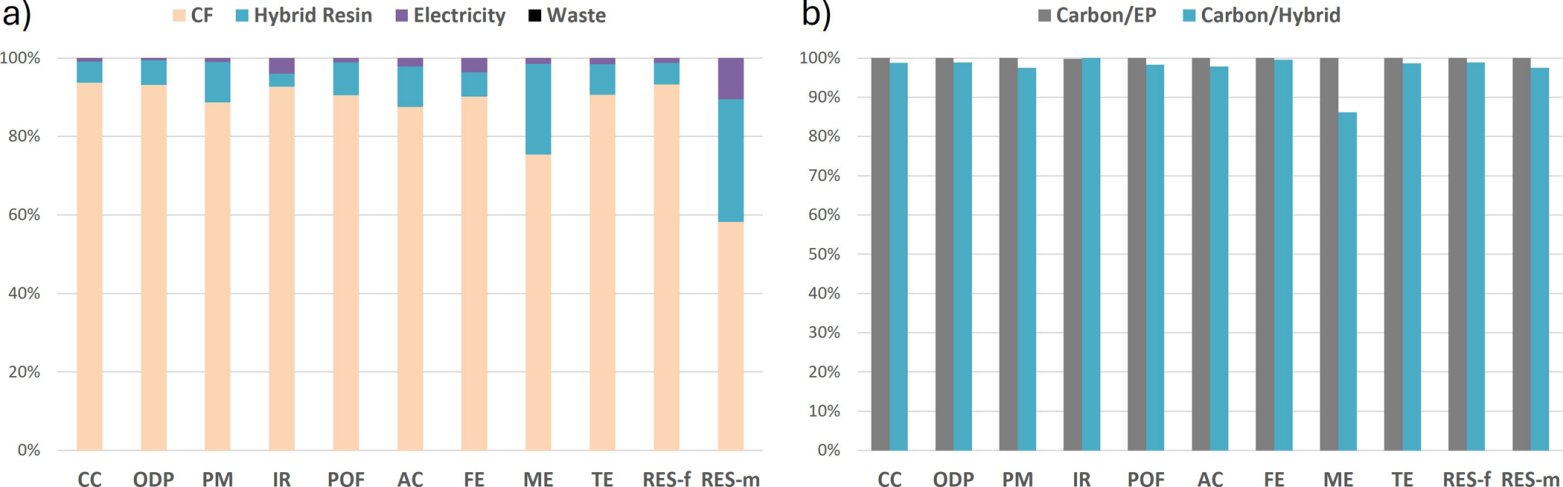
Distribution of each constituent of the carbon-based composites on the overall EI. (a) Carbon/hybrid and (b) comparison of the impacts of carbon/EP and carbon/hybrid composites.

Because CFs have the highest EI, a significant decrease of the environmental footprint of the composites cannot be expected from the resin substitution. The comparative results are displayed in Figure 6b. The hybrid-based CFRP demonstrates a reduction in all environmental indicators. Yet, this decrease is limited to 2–5%, except for marine eutrophication, which is reduced by 15%. The differences between the two CFRPs should not be considered sufficiently distinguishable to draw strict conclusions from this cradle-to-gate perspective.

In light of the previous results on neat resins and flax composites, a trend toward environmental impact improvements can still be highlighted. More importantly, the results strongly suggest that this emerging hybrid strategy is more promising and does not generate any detrimental environmental side effects.

### End-of-Life Management of Composites, a Material-Related Strategy

The waste management of composites, including biobased ones, remains a major issue.^20^ The most advanced recycling technologies are so far pyrolysis^52^ and solvolysis.^53^ It was demonstrated that the recovery of carbon fibers (rCF) was positive on both environmental and economic aspects compared to virgin CF.^54^ However, the recovery of glass fibers was, in most cases, not economically viable and not systematically beneficial for the environment.^20^

Covalent adaptable networks offer an alternative to conventional epoxy resin composite materials. First, the network’s dynamic behavior enables the possibility of reshaping and mechanically recycling the material. Second, in the present case study, a chemical pathway involving acetic acid (HAc) was confirmed to cleave the resin at low temperatures within a few hours.^27^ The process was effective for flax and carbon fibers and demonstrated no chemical or mechanical degradation of both fiber types.^27,28^ However, the environmental benefits of recovering low EI flax fibers need to be discussed.

The LCA was performed in a cradle-to-grave approach, as shown in Tables S12–S14. A virgin material was considered at first, identical in all scenarios. In the reference scenarios, when decommissioned, the composite is landfilled or incinerated (for flax), and a new virgin material is produced. An EP-based composite with landfill EoL management was used as a reference. For the chemical recycling scenario, the first virgin material is depolymerized, and the recovered fibers are used with a virgin matrix to produce a new composite with a quality factor of 0.7. For the thermo-mechanical recycling scenario, we assumed that composite plates could be cut into large pieces and reshaped into semistructural parts by welding them and exploiting the dynamic network, similarly to the mechanical recycling of thermoplastic materials. For example, a meter-square turbine blade could be reshaped into sports equipment or fiberboard-like materials. It remains important to note that mechanical recycling generally lowers the quality of the materials. Moreover, regulations and safety often limit recycled products to lower added-value applications. High-performance materials from mechanical recycling remain, at the current stage of development, difficult to envision,^20^ while chemical recycling is peaking up in speed but are still facing difficulties to become economically viable.^20,54^ Yet, the regulatory pressure over banning landfill, and the gates fee (around 150–200€/ton)^32^ tends to facilitate the adoption of more circular practices.

The LCA was first performed on CFRP (Figure 7). As observed in the previous section, a relatively positive outcome arises from the use of the hybrid instead of the EP when a conventional landfill EoL is considered. The two scenarios were estimated to yield around 85 kgCO_2_eq with a fossil resource depletion equivalent to 1540 MJ. High levels of eutrophication and acidification were also caused by the landfill. Detailed results are given in Table S18. As expected, mechanical recycling revealed the best environmental savings with a drastic reduction of about 20–25% of all indicators except eutrophication, reducing the CC indicator to 68 kgCO_2_eq and the fossil resource depletion to 1186 MJ. These values for two cumulative applications are only 10% superior to the production of a single virgin material. Although some parameters such as collecting, sorting, and transportation are neglected between both applications, they give promising results toward the direct reuse of CFRP based on the hybrid resin and could justify the interest of these new matrices from the perspective of the entire life cycle.

**Figure 7:**
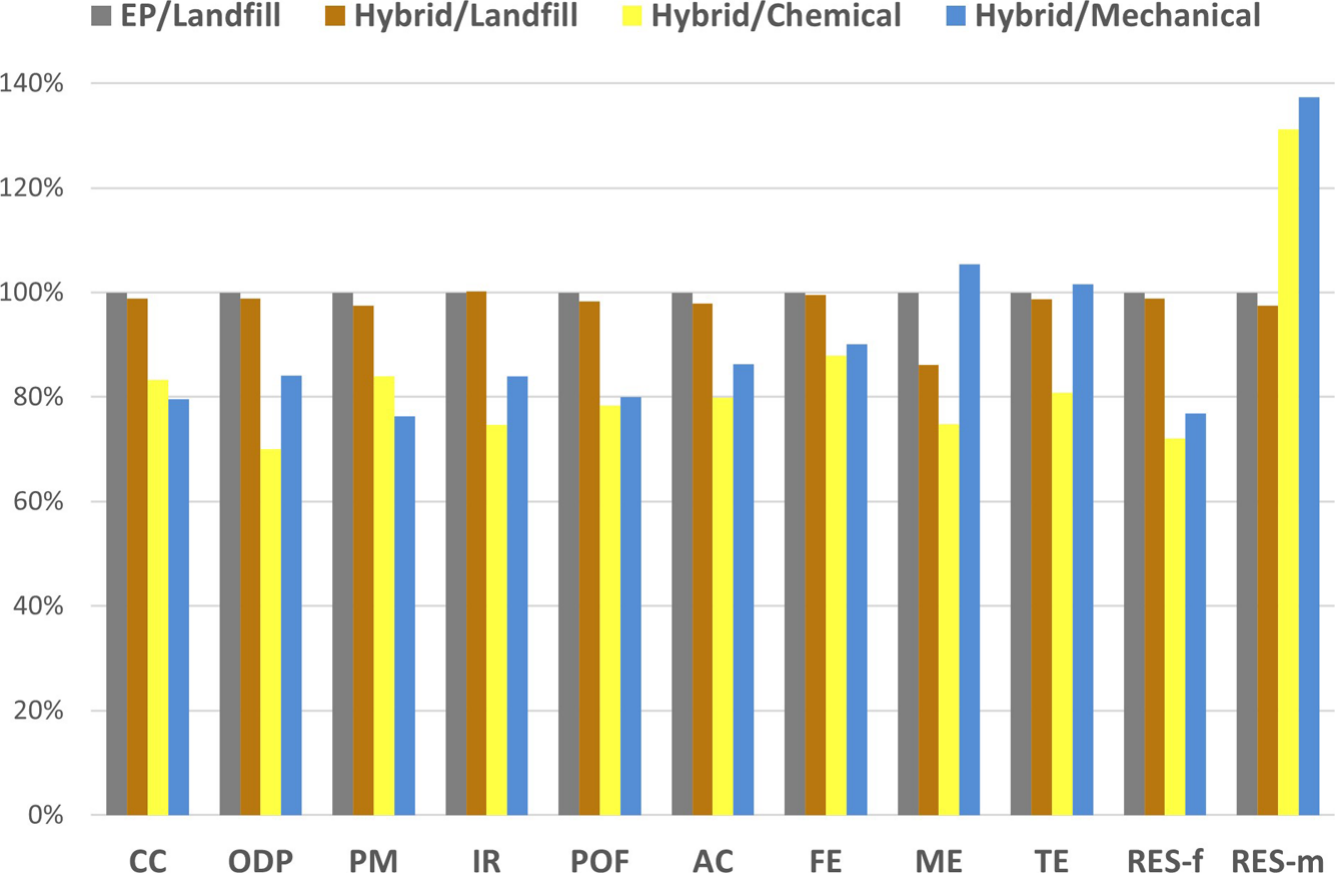
Life cycle assessment for the different EoL scenarios exploiting the hybrid matrices with carbon fiber reinforcement. Results are normalized to the EP-based carbon composite, a reference, with landfill EoL.

The chemical recycling process also revealed positive outcomes compared to the landfill scenario, with a reduction of the CC effect by 17% and acidification or eutrophication by about 20%. The fossil resource depletion was reduced by 28% due to virgin CF production avoided and was also lower than that of mechanical recycling. It is important to note that the reuse of HAc was considered in the loop, which is critical for affording the benefits of chemical recycling. Further discussion on that point can be found in the dedicated section for improvements.

Chemical recycling shows only a slightly higher EI than mechanical recycling. Typically, solvolysis presents a higher environmental impact than mechanical recycling,^20,54^ but it yields higher quality fibers that can be considered in advanced applications with greater economic values. The results indicate that oxidative chemical recycling can be considered an environmentally sound EoL scenario for CFRP.

The interest in recycling materials lies in the balance between the environmental cost of producing virgin materials and the environmental cost of the recycling processes.^20^ For materials with high EI and economic value such as CFRP or metallic structures,^55,56^ the recycling process tends to systematically give lower EI than virgin material production. For materials with low EI, such as NF-based composites, balancing the recycling processes with the virgin materials is more challenging,^57^ and to date, the recovery of flax fibers remains technically challenging and has never been environmentally assessed.

The results for complete life cycle scenarios for flax-composites with different EoL options are presented in Figure 8. Similar to the cradle-to-gate analysis, the flax/hybrid composite demonstrates a 15% CC and RES-f decrease compared with the EP-based one. Unlike CFRP, incineration with energy recovery was considered as the NFs can burn, unlike glass and carbon fibers, which must be removed and treated separately. The incineration can be advantageous as it is a rapid technique suitable for all types of NF/polymer matrix combinations because all materials can combust.^57^ However, the results showed that the recovered energy does not balance with the total process and leads to a 28% increase in CC and water eutrophication. Other indicators tend to be similar to the reference case scenario but are higher than the landfill of the flax/hybrid composite. The higher GWP can be understood through the CO_2_ and other combustion gases emitted during burning. As the European electricity mix is not a large contributor to GWP, using waste as an energy source is not advantageous. The loss of materials with only the energy recovery through a greenhouse gases emitting process does not appear to be the best choice.^20^

**Figure 8:**
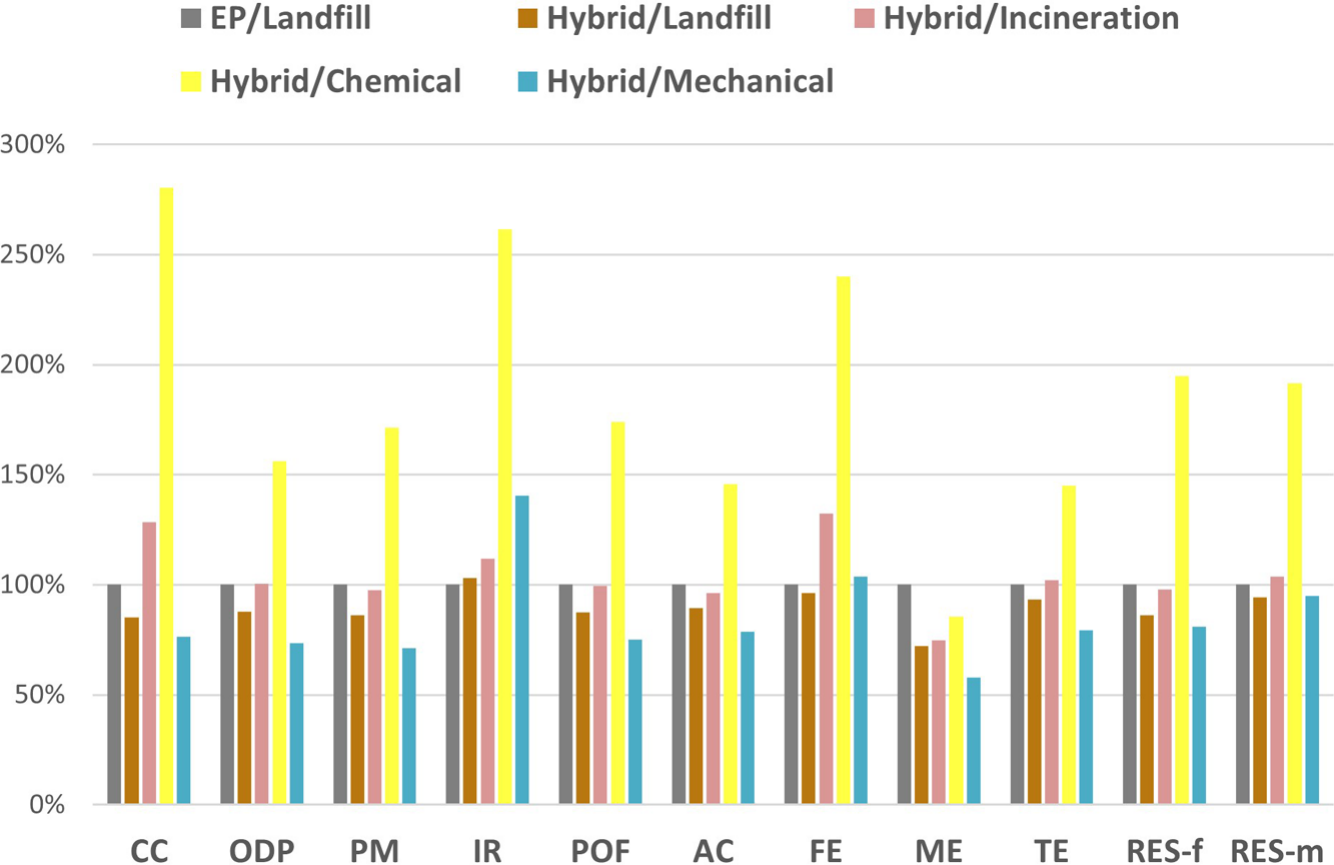
Life cycle assessment for the different EoL scenarios exploiting hybrid matrices with flax fiber reinforcement. Results are normalized to the EP-based flax composite, reference, with landfill EoL.

Finally, chemical and mechanical recyclings were evaluated. The chemical depolymerization to recover flax fibers is the worst scenario. The process is a low consumer of energy as the temperature required to perform the depolymerization is low (below 65 °C), and reagents are water, HAc, and hydrogen peroxide, which are typically considered environmentally friendly.^58^ However, while a positive outcome might have been expected, the CC indicator increased by 180% compared to that of the reference. Fossil resource depletion increased by more than 90%. All other categories imparted a 50% higher impact, except for marine eutrophication, but did not provide significant differences. In that sense, the chemical recycling of NFC, under the current conditions, is not sustainable. The issue lies in the use of HAc and its production route (see Figure S8). About 80% of commercially available glacial acetic acid is produced from the carbonylation (carbon monoxide from syngas) of methanol under pressure (30–60 bar) and temperature (150–200 °C) in the presence of cobalt (BASF) or ruthenium (Monsanto) catalysts, and hydrogen iodide.^59^ The EI of petro-based acetic acid is, therefore, elevated (3.3 kgCO_2_eq/kg and 62 MJ/kg). The large quantities of acetic acid required to swell and cleave the network hamper the environmental benefits of recovering NFs, even when recirculating 90% HAc is considered.

Conversely, mechanical recycling appears more promising as a reduction of 24% of the CC indicator, and energy demand is observed. Except for ionizing radiation, all impacts are reduced by about 20–30%, demonstrating that NF, using CAN matrices, can provide substantial environmental benefits in composite materials. Indeed, CAN-based composites offer properties and processes similar to traditional thermosets while also enabling more sustainable recycling pathways.

### Discussions on Improvement Perspectives and Result Extrapolation

Based on the previous results, several pathways can be drawn to further reduce the overall environmental footprint of hybrid-based composites. Gaining energy consumption, management efficiency, and renewable electricity production would benefit all stages. However, this is more related to state policy than research laboratories and will not be further discussed. Moreover, it is important to note that the following study used market data and an energy mix from Europe. Market data includes a mix of European production and imported chemicals, picturing the current situation in the EU. The transportation footprint could be reduced in other parts of the world but often remains limited compared to sourcing and production, and energy could play a more important role. The energy mix of Europe has a lower CO_2_ footprint compared to the world average^60^ (292 vs 486 gCO_2_eq/kWh). Similar studies in the US (386 gCO_2_eq/kWh) or Asia (594 gCO_2_eq/kWh) would probably increase the overall footprint, but many factors should also be considered and situations should be considered independently.

Regarding the production of cyclic carbonates, the process was considered using supercritical CO_2_. When using scCO_2_, epoxy is the major contributor to the cyclic carbonate EI, accounting for nearly 80% of all indicators. Hence, reducing the EI of the starting epoxy is the key to reducing the cyclic carbonate footprint. For epoxy, the major contributors are the epichlorohydrin and the phenol/polyol backbone. NaOH also represents a non-negligible share but will not be discussed in the current work. Depending on the backbone, ECH can be the major contributor, such as in TMPTGE (Figure S1), or second such as RDGE (Figure S2). The inputs in the current studies were taken from the current market and, therefore, petro-based. However, large efforts have been performed during the last decades to establish more sustainable pathways toward epoxy through the use of biobased epichlorohydrin^61^ and renewable precursors^62^ with significant benefits. The use of biobased ECH, derived from glycerol as a biofuel byproduct, displayed a reduction of the CC indicator by 60%.^63^ Bio-ECH is commercialized under the trademark Epicerol and appears to be a reliable pathway to reduce the EI of both epoxy and cyclic carbonates (see Figure S6). A reduction of between 20 and 30% of the CC effect can be expected for both TMPTGE and TMPTC, as shown in Figures 9 and S7.

**Figure 9:**
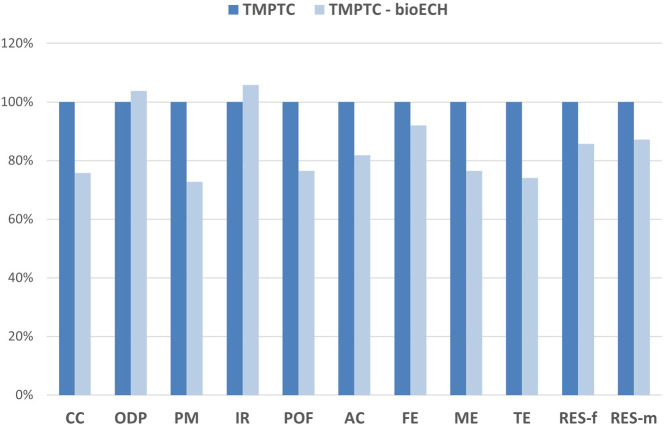
Environmental interest of replacing petro-based ECH with glycerol-derived ECH for TMPTC.

Additionally, CO_2_-sourcing could play a critical role in the future of PHU’s environmental footprint. While major efforts are being pushed to increase carbon capture environmental benefits,^64,65^ the quantity of exploitable data for LCAs, as much as actual market availability for CO_2_ “green” sourcing, remains low. Currently, the vast majority of available CO_2_ on the market is obtained from direct capture in chemical (ammonia production), energy, and cement plants, limiting to some extent the emissions of additional CO_2_ in the atmosphere. Yet, exploring direct air carbon capture/removal^66^ and storage within cyclic carbonates could be a promising pathway toward net-zero carbon materials. However, more reliable data for LCA modeling^65^ and higher TRL are still expected to be relevant for industrial production scales and ensure positive outcomes.

It is important to note that resorcinol is a major contributor to the EI of RDGE and therefore the epoxy and hybrid resins. Indeed, resorcinol was chosen in the present work as it is often considered in the literature as a potential alternative to bisphenol A (BPA) and delivers low viscosity.^37,67^ Resorcinol can be bioderived from glucose by microbial processes. However, the input data come from the market, where resorcinol is produced from benzene. Based on the data currently available in Ecoinvent, resorcinol EI is almost three times more significant than BPA (Figure S5). A significant reduction of the EI might be expected from bioresorcinol but has not been assessed in the literature. The economic viability of bioresorcinol is not ensured. Moreover, resorcinol has been recently reported as a proven endocrine disruptor and listed as a substance of very high concern under REACH classifications.^68^ Therefore, future environmental improvements for synergetic hybridization should focus on low-viscosity epoxy, both phenolic and aliphatic, derived from renewable resources. The toxicity should be further considered, as well. As potential substitutes, furan-derived epoxy^69^ or cyclo-aliphatic alcohols derived from lignin^70^ could provide less toxic precursors with environmental gains.

Equally, amines account for around 20% of resins in terms of EI. In the case of reducing the EI contribution of epoxy and cyclic carbonates, they will become major contributors. Biobased amines are facing a rising interest in both industries and academia.^35^ However, to date, there is no data available on the environmental outcomes of biobased amines, with the exception of biobased aniline, proven better than its petro-sourced counterpart.^71^ Reducing the EI of the curing amines through the use of biobased precursors from lignin or sugars would benefit the composite industry.

The energy required to cure resins typically contributes to 10–15% of all indicators for EP and hybrid EP-PHU, and 30–40% in the case of the more energy-demanding pure PHU. These results highlight the importance of optimized curing processes and exploring fast-curing chemistries to minimize the contributions at this stage. Fast-curing epoxy and PHUs could help significantly reduce energy demand by lowering the curing temperature and time while also providing improved production rates. However, the LCA should model the synthesis of more reactive building blocks accurately to avoid upstream environmental burdens. Similarly, the exploration of catalytic systems^72^ for PHUs and epoxy, including light-cured systems,^73,74^ could be a promising pathway toward low-energy composite materials. However, these strategies require further development before reaching composite applications and providing an accurate model in LCA. For instance, the in-depth penetration of light in the composite thickness remains a technical challenge.^75^ Moreover, the catalytic system should also be modeled in LCA software, as it is most likely to be unavailable in a common database, and other aspects such as toxicity and aging should be considered. Nevertheless, future work in that direction might leverage further benefits.

Finally, recycling was pointed out to have an important effect on the overall LCA. Mechanical recycling enabled by the hybrid’s CAN nature affords substantial EI reductions compared to nonrecyclable matrices. However, the significant reduction in properties limits their application to lower-value uses. Therefore, finding ways to recover both the matrix and the fibers would still be beneficial. The relevance of recovering NFs, in light of the current results, must be considered from both environmental and economic perspectives.

The current chemical degradation process gives room for large improvements. The results indicate that market-grade HAc is unsustainable. The current process was extrapolated from lab-scale experiments. In that sense, reducing the quantity of degradation solution could help reduce the consumption of all reagents. Moreover, glacial acetic acid (>99%) is currently used. Reducing the concentration of acetic acid (HAc) below 20% would allow for the use of white vinegar, which is biobased acetic acid derived from ethanol fermentation. To investigate this possibility, a quick experiment was performed, see Figure S9, and the EI was modeled. It is important to note that a higher amount of hydrogen peroxide was necessary. Longer times and higher temperatures were also required (15 h versus 4 h, at 70 °C instead of 60 °C). As biobased HAc or vinegar is not available in the EcoInvent database, vinegar was replaced by a water/ethanol solution (20% concentration). The enhanced process demonstrates a reduction of 80% of CC and 70% of fossil resource depletion, as shown in Figure 10. Despite the significant decrease in the EI for the chemical degradation process, which would also become more eco-friendly to recover carbon fibers (see Figure S10), the production of 1 kg of virgin flax fibers remains less detrimental to the environment than 1 kg of recycled flax fibers. Therefore, alternative pathways must be considered to design sustainable EoL scenarios for NF composites. The valorization of the currently wasted matrices would be highly beneficial to access an eco-friendly process for retrieving NFs. Moreover, directly reusing the depolymerization solution for multiple recycling batches may also be beneficial. At the current stage, the chemical recycling of NFs does not seem to be an ideal pathway. The use of vinegar/biobased acetic acid in addition to not providing the expected environmental benefits from recycling might lead to a less economically viable process. Further scientific efforts toward environmentally friendly recycling processes are still needed and should consider the thermal sensitivity of NFs (i.e., process below 150 °C) to be suited, adding further to the technical challenge.

**Figure 10:**
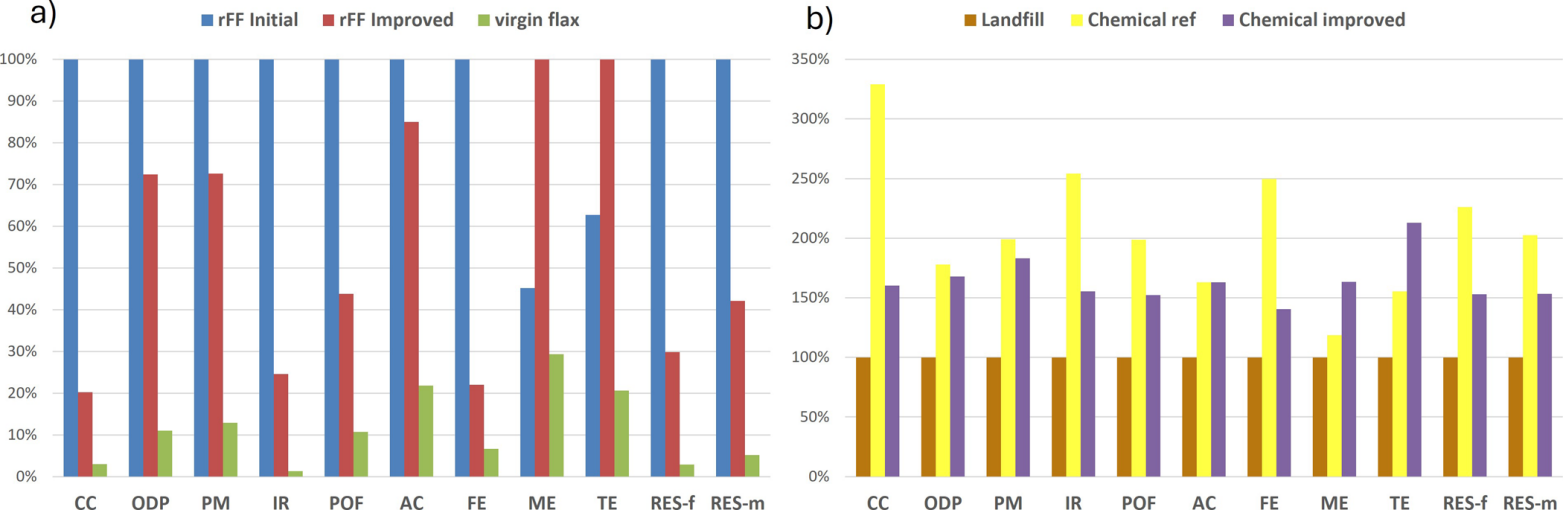
Strategy to improve chemical recycling through the use of vinegar to replace petro-based HAc. (a) Comparison of 1 kg flax fibers from the initial chemical recycling process, the improved one, and virgin flax production, (b) Comparison of the LCA outcomes from the chemical recycling of flax/hybrid composites.

## Conclusions

The environmental footprint of carbon and flax fiber composites was assessed and compared when using as matrices an EP, a PHU, or a scalable and efficient CAN arising from EP-PHU hybrids.

The results in this work indicate that cyclic carbonates, the PHU monomer, can provide positive environmental benefits compared with their epoxy precursors when produced from CO_2_ using supercritical conditions. However, the resulting polyhydroxyurethane and its subsequent composites cannot be considered as environmentally beneficial compared to an epoxy network. This is ascribed to the detrimental effect of the more energy-intense protocol required to cross-link PHUs. Still, the carbonation process does not impart environmental burden and both EP and PHU present similar results.

The synergetic EP-PHU hybrid CAN, whose processing and properties are more suited to the composite industry, exhibits substantial benefits. Through more efficient curing, the environmental benefit of cyclic carbonate production is retained. When combined with flax fibers, a reduction of about 15% of the global warming potential and fossil energy can be expected compared with epoxy matrices. The use of carbon fibers, which is more impactful, reduces the interest in using hybrid EP-PHU from cradle-to-gate perspectives with only 2–5% environmental gains.

When considering the end-of-life of the composites, the mechanical and chemical recycling of carbon fiber composites with the CAN resin is demonstrated to be environmentally valuable. Only marginal gain arises from mechanical recycling compared to that from chemical. Given the higher economic value of chemically recycled carbon fibers, oxidative chemical depolymerization could be a promising pathway to efficiently retrieve carbon fibers, provide environmental benefits, and reuse them in secondary applications, even though the polymer matrix is wasted.

The recycling of NF composites is more intricate to balance. The mechanical recycling, accessible through the synergetic EP-PHU hybrid CAN, is demonstrated as beneficial. The low environmental impact of the composite makes the chemical recycling process not competitive, with an increase of 300% of the climate change indicator compared to a virgin composite. This high impact is ascribed to the negative environmental footprint of acetic acid. The decrease in the acetic acid concentration opens the door to the use of biobased acetic acid (vinegar), which helps reduce the impact of chemical recycling by 90% for NFs. However, it remains three times more impactful than virgin flax fiber production. Through the entire LCA, the use of vinegar to chemically recycle NF composites accounts for a 33% increase in environmental impact.

For NFs, developing the recycling and valorization of the matrices should be investigated in the future to expect a potentially positive outcome. Optimizing the dynamic behavior to enhance mechanical recycling and drastically reducing the environmental footprint of the matrix by using biobased precursors, such as bioepichlorohydrin, appears to be a more suitable approach to develop/create greener materials.

These results demonstrate that CANs and synergetic EP-PHU hybrid, in particular, ideally derived from renewable resources such as biomass and CO_2_, can provide a relevant pathway toward sustainable composites by reducing the material’s environmental footprint and opening new recycling strategies. Future technical and scientific work should focus on optimizing sourcing and recycling pathways to valorize all constituents but also investigate the long-term performances of CAN-based composite materials and structures made of them. The LCA should be further conducted on actual engineered structures, taking into account the use phase and therefore the mechanical properties. In particular, the use of dynamic networks, intrinsically repairable, with higher mechanical performance could extend significantly the lifetime of structures and, as a consequence, diminish the environmental footprint. A techno-economic assessment should also be performed to investigate the economic interest of such materials in the near future.

## Supplementary Material



## Notes

Additional experimental details, such as life cycle inventories, detailed environmental outputs, and complementary graphs of environmental indicators (PDF)
