# Supplementary material for: Can Polyhydroxyurethane-Derived Covalent Adaptable Networks Provide Environmental Benefits in Composite Applications?
Source: ACS Sustain Chem Eng. 2025 Jul 15;13(29):11238–53. doi: 10.1021/acssuschemeng.5c01260 (PMC12308879; doi:10.1021/acssuschemeng.5c01260)
Supplement: Supplementary file 1 [file sc5c01260_si_001.pdf]

Can polyhydroxyurethane-derived covalent adaptable networks provide environmental benefits in composite applications?

Guillem Seychal,*,†,‡ Pauline Bron,‡ Olivier Talon,¶ Nora Aramburu,‡ and Jean-Marie Raquez*,†,§

†*Laboratory of Polymeric and Composite Materials, Center of Innovation and Research in Materials and Polymers (CIRMAP), University of Mons, Place du Parc 23, Mons, 7000, Belgium*

‡*POLYMAT and Department of Advanced Polymers and Materials: Physics, Chemistry, and Technology, Faculty of Chemistry, University of the Basque Country UPV/EHU, Paseo Manuel de Lardizabal 3, Donostia-San Sebastián, 20018, Spain*

¶*Materia Nova, Avenue Copernic 3, Mons, 7000, Belgium*

§*WEL Research Institute, Wavre 1300, Belgium*

E-mail: guillem.seychal@umons.ac.be; jean-marie.raquez@umons.ac.be

Supporting Information

17 pages, 10 figures & 20 tables

Contents

Life Cycle Inventory **S5**

Monomer EI results **S12**

Epoxy synthesis and results S12

TMPTC synthesis S13

Resin detailed results **S14**

Composite detailed results **S15**

Full Life cycle assessment **S16**

Carbon fiber reinforced composites S16

Flax fiber reinforced composites S16

Discussions about improvements **S17**

Bisphenol A and resorcinol S17

Bio-epichlorohydrin interest S18

Impact of acidolysis and improvements S19

List of Figures

S1 TMPTGE synthesis, contribution analysis S12

S2 RDGE synthesis, contribution analysis S12

S3 Comparison of 1 kg of TMPTGE and 1 kg of TMPTC S13

S4 Comparative EI for TMPTC produced using supercritical CO_2 ($scCO_2$) and gaseous CO_2 in solvent (ethyl acetate).a) Contribution of the composition to the EI of TMPTC from solvent, and b) compared EI. S14

S5 Comparative EI between bisphenol-A (BPA) and resorcinol for 1 kg, obtained from EcoInvent 3.4 database. S17

S6 Comparison of EI of biobased (glycerol) and petro-based (allyl chloride) epichlorohydrin. S18

S7 Comparison of EI of TMPTGE from petro-based and biobased epichlorohydrin.S18

S8 EI of the chemical recycling process and importance of HAc. a) contribution of each constituent in the recycling process, and b) shares of HAc in the depolymerization solution mixture. S19

S9 Pictures of the recycling of the flax/hybrid composite in 20% acetic acid. . . S19

S10 Improvement of the chemical recycling processes for carbon fiber reinforced composite. Comparison of the environmental footprint of virgin carbon fibers (vCF) vs the glacial acetic acid process (rCF) and the vinegar-based process. S20

List of Tables

S1 Life Cycle Inventory of TEBAC S5

S2 Life Cycle Inventory the Raney Nickel S5

S3 Life Cycle Inventory of RDGE S6

S4 Life Cycle Inventory of TMPTGE S6

S5 Life Cycle Inventory of TMPTC S7

S6 Life Cycle Inventory of mXDA S7

S7 Life Cycle Inventory of carbon fibers S7

S8 Life Cycle Inventory of TMPTC, using solvent S8

S9 Life Cycle Inventory of biobased epichlorohydrin S8

S10 Life Cycle Inventories of the different resins under study (including curing) . S9

S11 Life Cycle Inventories of the different composites under study (including curing) S9

S12 Life Cycle Inventory of the chemical recycling S10

S13 Life Cycle Inventory of the recycled composites S11

S14 Life Cycle Inventory for incineration with energy recovery of flax fiber compositesS11

S15 EIA of TMPTGE, TMPTC from supercritical CO2 and TMPTC from CO2 in solvent. Results for 1 kg of monomer. S13

S16 Environmental impacts of the cured resins (1kg) S14

S17 Environmental impact assessment results flax fiber reinforced composite . . . S15

S18 Environmental impact assessment results carbon fiber reinforced composite . S15

S19 LCA results carbon fiber reinforced composite S16

S20 LCA results flax fiber reinforced composite S16

Life Cycle Inventory

Table S1: Life Cycle Inventory of TEBAC

Name	Input dataset / emitted substance	Input	Output	Unit
Production of benzyltriethylammonium chloride (TEBAC)		-	0.660	kg
Benzyl chloride	Benzyl chloride (RER) market	0.390	-	kg
Triethyl amine	Triethyl amine (GLO), market	0.283	-	kg
Ethyl acetate	Ethyl acetate (GLO), market	0.006	-	kg
Acetonitrile	Acetonitrile (GLO), market	0.018	-	kg
Hazardous waste, for incineration	Hazardous waste, for incineration (Europe without Switzerland), market	-	0.053	kg

Table S2: Life Cycle Inventory the Raney Nickel

Name	Input dataset / emitted substance	Input	Output	Unit
Production of Ni-Al Alloy	-	-	1	kg
Aluminum	Aluminum, primary, ingot (AI Area, EU27 & EFTA)	0.50	-	kg
Nickel	Nickel, Class 1 (GLO), market	0.50	-	kg
Production of Ni-Al powder 100 um	-	-	0.99	kg
NiAl Alloy	-	1	-	kg
Argon	Inert gas for discharge lamps (GLO), market	0.33	-	kg
Electricity	Electricity, medium voltage (RER), market	0.75	-	kWh
Electricity	Electricity, medium voltage (RER), market	0.79	-	kWh
Production of Raney Nickel	-	-	0.40	kg
NiAl powder 100 um	-	1	-	kg
Sodium hydroxide	Sodium hydroxide, without water, in 50% solution state RER, market	0.80	-	kg
Water	Water, deionised (Europe without Switzerland), market	68.20	-	kg

Table S3: Life Cycle Inventory of RDGE

Name	Input dataset / emitted substance	Input	Output	Unit
Production of RDGE		-	1	kg
Resorcinol	Resorcinol (GLO), market	0.500	-	kg
Epichlorohydrin	Epichlorohydrin (GLO), market	0.830	-	kg
Sodium hydroxide	Sodium hydroxide, without water, in 50% solution state (RER), market	0.730	-	kg
Water	Water, cooling, unspecified natural origin, RER	8E-3	-	m3
Water	Water, river, RER	5E-4	-	m3
Water	Water, well, RER	4E-4	-	m3
Electricity	Electricity, medium voltage (RER), market	0.224	-	kWh
Heat	Heat, district or industrial, natural gas (RER), market	1.059	-	MJ
Heat	Heat, from steam, in chemical industry (RER), market	0.117	-	MJ
Emissions in air, water	Water/m3, RER	-	0.001	m3
Emission in water, chloride	Chloride	-	0.328	kg
Emission in water, Sodium	Sodium (I)	-	0.230	kg
Emission in water, water	Water, RER	-	4E-4	m3
Wastewater	Wastewater, average (CH), market	-	3E-8	m3
Wastewater	Wastewater, average (Europe without Switzerland), market	-	1E-6	m3

Table S4: Life Cycle Inventory of TMPTGE

Name	Input dataset / emitted substance	Input	Output	Unit
Production of TMPTGE		-	1	kg
Trimethylolpropane	Pentaerythritol (GLO), market	0.440	-	kg
Epichlorohydrin	Epichlorohydrin (GLO), market	0.920	-	kg
Sodium hydroxide	Sodium hydroxide, without water, in 50% solution state (RER), market	0.790	-	kg
TEBAC	TEBAC	.04	-	kg
Water	Water, cooling, unspecified natural origin, RER	8E-3	-	m3
Water	Water, river, RER	5E-4	-	m3
Water	Water, well, RER	4E-4	-	m3
Electricity	Electricity, medium voltage (RER), market	0.224	-	kWh
Heat	Heat, district or industrial, natural gas (RER), market	1.059	-	MJ
Heat	Heat, from steam, in chemical industry (RER), market	0.117	-	MJ
Emissions in air, water	Water/m3, RER	-	0.001	m3
Emission in water, chloride	Chloride	-	0.361	kg
Emission in water, Sodium	Sodium (I)	-	0.397	kg
Emission in water, water	Water, RER	-	4E-4	m3
Wastewater	Wastewater, average (CH), market	-	3E-8	m3
Wastewater	Wastewater, average (Europe without Switzerland), market	-	1E-6	m3

Table S5: Life Cycle Inventory of TMPTC

Name	Input dataset / emitted substance	Input	Output	Unit
Production of TMPTC	-	-	1.58	kg
TMPTGE	TMPTGE	1.10	-	kg
Carbon Dioxide	Carbon dioxide, liquid (RER), market	0.50	-	kg
TEBAC	TEBAC	0.04	-	kg
Electricity	Electricity, medium voltage (RER), market	3.30	-	kWh

Table S6: Life Cycle Inventory of mXDA

Name	Input dataset / emitted substance	Input	Output	Unit
Production of m-xylylene diamine (mXDA), from[1]	-	-	1.00	ton
Xylene	Xylene (RER), market	1.61	-	ton
Ammonia	Ammonia, anhydrous, liquid (RER), market	0.61	-	ton
Dioxygen	Oxygen, liquid (RER), market	1.45	-	ton
Dihydrogen	Hydrogen, gaseous(GLO), market	0.07	-	ton
N-methylpyrrolidone	N-methyl-2-pyrrolidone (GLO), market	0.13	-	ton
Raney Nickel	Raney Nickel	0.01	-	ton
Electricity	Electricity, medium voltage (RER), market	40.60	-	MJ

Table S7: Life Cycle Inventory of carbon fibers

Name	Input dataset / emitted substance	Input	Output	Unit
Production of carbon fibers[2]	-	-	1	kg
Acrylonitrile	Acrylonitrile (GLO), market	2.09	-	kg
Vinyl Acetate	Vinyl acetate (GLO), market	0.02	-	kg
Nitrogen	Nitrogen, liquid (RER), industrial	11.50	-	kg
Steam	Steam, in chemical industry (RER), market	33.90	-	kg
Heat	Heat, district or industrial, natural gas (Europe without Switzerland), industrial furnace >100kW	360.20	-	MJ
Electricity	Electricity, medium voltage (RER), market	33.40	-	kWh

Table S8: Life Cycle Inventory of TMPTC, using solvent

Name	Input dataset / emitted substance	Input	Output	Unit
Production of TMPTC at low pressure using solvent	-	-	388	g
TMPTGE	TMTPGE	300	-	g
CO2	Carbon dioxide, liquid (RER), market	131	-	g
Ethyl Acetate (solvent)	Ethyl acetate (GLO), market	135	-	g
Water	Water, deionised (Europe without Switzerland), market	400	-	g
NaCl	Sodium chloride, brine solution (GLO), market	10	-	g
MgSO4	Magnesium sulfate (GLO), market	20	-	g
Ethylene bromide (catalyst)	Ethylene bromide (RER), market	9.6	-	g
Electricity	Electricity, medium voltage (RER), market	0.08	-	kWh
Heat	Heat, district or industrial, natural gas RoW, market	0.04	-	MJ
Solvent waste	Spent solvent mixture (Europe without Switzerland), market	-	545	g
Hazardous waste	Hazardous waste, for incineration (Europe without Switzerland), market	-	20	g

Table S9: Life Cycle Inventory of biobased epichlorohydrin

Name	Input dataset / emitted substance	Input	Output	Unit
Production of bio-epichlorohydrin (bio-ECH) Almena 2016	-	-	1	ton
Glycerol	Glycerine FR, treatment of used vegetable cooking oil, purified, esterification , Cut-off, U	1.250	-	ton
Methanol	Methanol (RER), market	0.235	-	ton
HCl	Hydrochloric acid, without water, in 30% solution state (RER), market	1.104	-	ton
NaOH	Sodium hydroxide, without water, in 50% solution state (RER), market	0.573	-	ton
Water	Water, well	4070	-	L
Heat (Natural Gas)	Heat, district or industrial, natural gas (RER), market	8.640	-	MJ

Table S10: Life Cycle Inventories of the different resins under study (including curing)

Name	Input dataset / emitted substance	Input	Output	Unit
Epoxy resin, cured	-	-	1.00	kg
RDGE	RDGE	0.63	-	kg
mXDA	mXDA	0.37	-	kg
Cure (press) 0.5 h 80 °C	Electricity, low voltage (RER), market	1.00	-	kWh
Post-cure (oven) 1h 160 °C	Electricity, low voltage (RER), market	0.40	-	kWh
PHU Resin, cured	-	-	1.00	kg
TMPTC	TMPTCscCO2	0.72	-	kg
mXDA	mXDA	.28	-	kg
Cure (press) 2 h 80 °C	Electricity, low voltage (RER), market	5.60	-	kWh
Cure (oven) 1 h 100 °C	Electricity, low voltage (RER), market	0.60	-	kWh
Post-cure (oven) 1h 150 °C	Electricity, low voltage (RER), market	0.20	-	kWh
Hybrid Resin, cured	-	-	1.00	kg
RDGE	RDGE	0.335	-	kg
TMPTC	TMPTC	0.335	-	kg
mXDA	mXDA	0.33	-	kg
Cure (press) 0.5 h 80 °C	Electricity, low voltage (RER), market	1.00	-	kWh
Post-cure (oven) 1h 160 °C	Electricity, low voltage (RER), market	0.40	-	kWh

Table S11: Life Cycle Inventories of the different composites under study (including curing)

Name	Input dataset / emitted substance	Input	Output	Unit
Flax fiber composite	-	-	1.00	kg
Resin formulation	Resin formulation (without energy)	0.44	-	kg
Flax	Fibre, flax, long, scutched (RER), market	0.66	-	kg
Curing	Electricity, low voltage (RER), market	1.40	-	kWh
Inert waste	Inert waste (RER), market	-	0.10	kg
Carbon fiber composite	-	-	1.00	kg
Resin formulation	Resin formulation (without energy)	0.33	-	kg
Carbon fiber	Carbon fiber	0.77	-	kg
Curing	Electricity, low voltage (RER), market	1.40	-	kWh
Inert waste	Inert waste (RER), market	-	0.10	kg

Table S12: Life Cycle Inventory of the chemical recycling

Name	Input dataset / emitted substance	Input	Output	Unit
Chemically recycled flax fibers	-	-	0.59	kg
Discarded Flax/Hybrid composite	Flax fiber composite	1.00	-	kg
Acetic Acid	Acetic acid, without water, in 98% solution state (GLO), market	16.00	-	L
Hydrogen peroxide	Hydrogen peroxide, without water, in 50% solution state (RER), market	1.20	-	L
Water	Water, deionised (Europe without Switzerland), market	2.80	-	L
Recycled acetic acid	Acetic acid, without water, in 98% solution state (GLO), market	-14.40	-	L
Electricity	Electricity, low voltage (RER), market	0.10	-	kWh
Electricity	Electricity, low voltage (RER), market	0.30	-	kWh
Waste	Spent solvent mixture (Europe without Switzerland), treatment	-	6.10	L
Chemically recycled carbon fibers	-	-	0.67	kg
Discarded Carbon/Hybrid composite	Carbon fiber composite	1.00	-	kg
Acetic Acid	Acetic acid, without water, in 98% solution state (GLO), market	16.00	-	L
Hydrogen peroxide	Hydrogen peroxide, without water, in 50% solution state (RER), market	1.20	-	L
Water	Water, deionised (Europe without Switzerland), market	2.80	-	L
Recycled acetic acid	Acetic acid, without water, in 98% solution state (GLO), market	-14.40	-	L
Electricity	Electricity, low voltage (RER), market	0.10	-	kWh
Electricity	Electricity, low voltage (RER), market	0.30	-	kWh
Waste	Spent solvent mixture (Europe without Switzerland), treatment	-	6.10	L
Chemical recycling from vinegar	-	-	Same	kg
Discarded composite	composite	1.00	-	kg
Water	Water, well	8	-	L
Biobased acetic acid	Ethanol, without water, in 99.7% solution state, from fermentation (GLO), market	2.00	-	L
Hydrogen peroxide	Hydrogen peroxide, without water, in 50% solution state (RER), market	4.00	-	L
Electricity	Electricity, low voltage (RER), market	1.00	-	L
Waste	Wastewater, average (Europe without Switzerland), market	-	2.50	L

Table S13: Life Cycle Inventory of the recycled composites

Name	Input dataset / emitted substance	Input	Output	Unit
Mechanically recycled composite	-	-	1.00	kg
Virgin composite	Carbon or flax composite	0.50	-	kg
Cutting	Electricity, low voltage (RER), market	0.30	-	kWh
Reshaping	Electricity, low voltage (RER), market	2.90	-	kWh
Waste	Inert waste (RER), market	-	0.50	kg
Composite from recycled carbon fibers	-	-	1.00	kg
recycled fibers	rCF	0.60	-	kg
Resin hybrid EP-PHU	Resin, without curing	0.35	-	kg
Curing	Electricity, low voltage (RER), market	1.40	-	kWh
Composite from recycled flax fibers	-	-	1.00	kg
recycled fibers	rFF	0.55	-	kg
Resin hybrid EP-PHU	Resin, without curing	0.35	-	kg
Curing	Electricity, low voltage (RER), market	1.40	-	kWh

Table S14: Life Cycle Inventory for incineration with energy recovery of flax fiber composites

Name	Input dataset / emitted substance	Input	Output	Unit
Incinerated flax fiber composite	-	-	0.00	kg
Virgin composite	Flax fiber composite	1.7	-	kg
Waste Incineration	Hazardous waste, for incineration (Europe without Switzerland), with energy recovery	-	1.70	kg

Monomer EI results

Epoxy synthesis and results

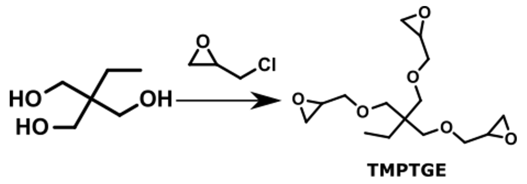

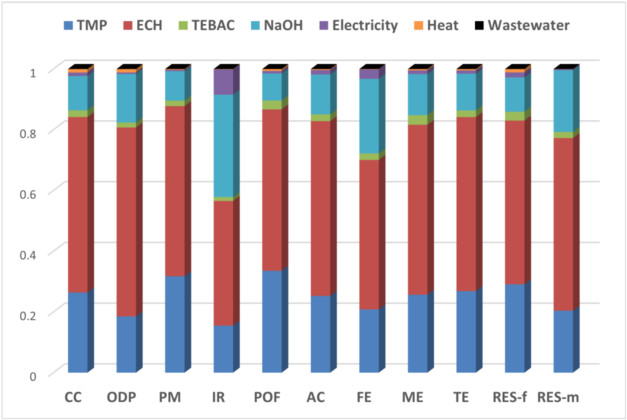

Figure S1: TMPTGE synthesis, contribution analysis

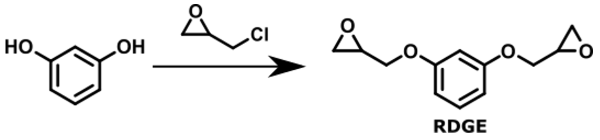

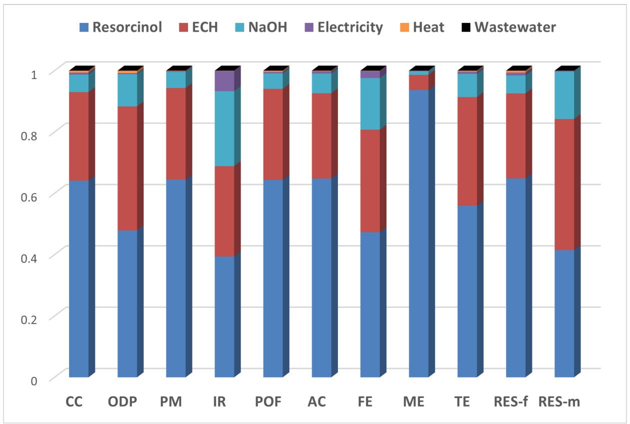

Figure S2: RDGE synthesis, contribution analysis

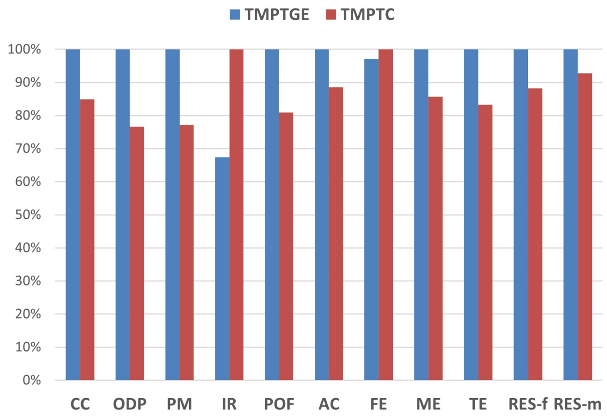

Figure S3: Comparison of 1 kg of TMPTGE and 1 kg of TMPTC

TMPTC synthesis

Table S15: EIA of TMPTGE, TMPTC from supercritical CO2 and TMPTC from CO2 in solvent. Results for 1 kg of monomer.

Indicator	Unit	TMPTGE	TMPTC ($scCO_2$)	TMPTC (solvent)
CC	kg CO2 eq	6.2	5.3	9.1
ODP	kg CFC11 eq	$2.6x10^{-7}$	$1.96x10^{-7}$	$2.5x10^{-7}$
PM	disease inc.	$2.7x10^{-7}$	$2.07x10^{-7}$	$3.2x10^{-7}$
IR	kBq U-235 eq	0.57	0.85	0.61
POF	kg NMVOC eq	0.02	0.02	0.02
AC	mol H+ eq	0.02	0.02	0.03
FE	kg P eq	0.002	0.002	0.003
ME	kg N eq	0.005	0.004	0.006
TE	mol N eq	0.05	0.04	0.06
RES-f	MJ	103	91	119
RES-m	kg Sb eq	$5.1x10^{-5}$	$4.8x10^{-5}$	$5.5x10^{-5}$
WAT	m3 depriv.	2.1	1.7	2.4
LU	Pt	20	18	22
HT-nc	CTUh	$5.6x10^{-8}$	$5.9x10^{-8}$	$8.3x10^{-8}$
HT-c	CTUh	$3.0x10^{-8}$	$2.4x10^{-8}$	$3.0x10^{-8}$
FWT	CTUe	168	122	166

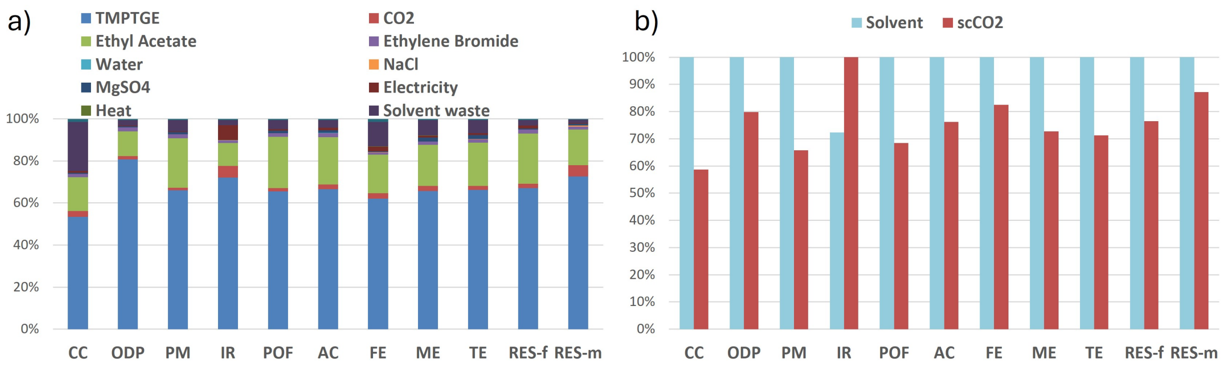

Figure S4: Comparative EI for TMPTC produced using supercritical CO_2 ($scCO_2$) and gaseous CO_2 in solvent (ethyl acetate).a) Contribution of the composition to the EI of TMPTC from solvent, and b) compared EI.

Resin detailed results

Table S16: Environmental impacts of the cured resins (1kg)

Indicator	Unit	EP	PHU	Hybrid
CC	kg CO2 eq	10.4	9.0	8.5
ODP	kg CFC11 eq	3.3x10^{-7}	2.6x^{-7}	2.8x^{-7}
PM	disease inc.	3.9x10^{-7}	2.6x10^{-7}	3.1x10^{-7}
IR	kBq U-235 eq	1.0	2.2	1.1
POF	kg NMVOC eq	0.04	0.03	0.03
AC	mol H+ eq	0.05	0.04	0.04
FE	kg P eq	0.003	0.004	0.003
ME	kg N eq	0.04	0.00	0.02
TE	mol N eq	0.07	0.06	0.06
RES-f	MJ	190	163	160
RES-m	kg Sb eq	6.4x10^{-5}	7.9x10^{-5}	6.2x10^{-5}
WAT	m3 depriv.	3.9	3.5	3.5
LU	Pt	25	28	22
HT-nc	CTUh	7.2x10^{-8}	8.9x10^{-8}	6.8x10^{-8}
HT-c	CTUh	5.9x10^{-8}	2.8x10^{-8}	4.2x10^{-8}
FWT	CTUe	208	105	156

Composite detailed results

Table S17: Environmental impact assessment results flax fiber reinforced composite

Indicator	Unit	Flax/EP	Flax/PHU	Flax/Hybrid
CC	kg CO2 eq	5.5	5.4	4.7
ODP	kg CFC11 eq	$1.8 x 10^{-7}$	$1.6 x 10^{-7}$	$1.6 x 10^{-7}$
PM	disease inc.	$2.4 x 10^{-7}$	2.0x10-1	$2.1 x 10^{-7}$
IR	kBq U-235 eq	0.64	1.73	0.66
POF	kg NMVOC eq	0.02	0.02	0.02
AC	mol H+ eq	0.03	0.04	0.03
FE	kg P eq	0.002	0.003	0.002
ME	kg N eq	0.02	0.01	0.02
TE	mol N eq	0.06	0.07	0.06
RES-f	MJ	96	106	83
RES-m	kg Sb eq	$3.6 x 10^{-5}$	$5.4 x 10^{-5}$	$3.45 x 10^{-5}$
WAT	m3 depriv.	1.9	2.0	1.8
LU	Pt	222	228	221
HT-nc	CTUh	$8.7 x 10^{-8}$	$1.09 x 10^{-7}$	$8.5 x 10^{-8}$
HT-c	CTUh	$3.1 x 10^{-8}$	$1.95 x 10^{-8}$	$2.4 x 10^{-8}$
FWT	CTUe	112	70	89

Table S18: Environmental impact assessment results carbon fiber reinforced composite

Indicator	Unit	Carbon/EP	Carbon/Hybrid
CC	kg CO2 eq	50.4	49.8
ODP	kg CFC11 eq	$1.5 x 10^{-6}$	$1.4 x 10^{-6}$
PM	disease inc.	$1.0 x 10^{-6}$	$9.8 x 10^{-7}$
IR	kBq U-235 eq	7.4	7.5
POF	kg NMVOC eq	0.11	0.11
AC	mol H+ eq	0.13	0.13
FE	kg P eq	0.012	0.011
ME	kg N eq	0.03	0.03
TE	mol N eq	0.24	0.23
RES-f	MJ	907	897
RES-m	kg Sb eq	$6.0 x 10^{-5}$	$5.9 x 10^{-5}$
WAT	m3 depriv.	11.5	11.4
LU	Pt	65	64
HT-nc	CTUh	$1.6 x 10^{-7}$	$1.6 x 10^{-7}$
HT-c	CTUh	$9.8 x 10^{-8}$	$9.2 x 10^{-8}$
FWT	CTUe	149	131

Full Life cycle assessment

Carbon fiber reinforced composites

Table S19: LCA results carbon fiber reinforced composite

Indicator	Unité	Reference	Landfill	Chemical	Mechanical
CC	kg CO2 eq	85.6	84.7	71.4	68.3
ODP	kg CFC11 eq	2.5x10^{-6}	2.5x10-3	1.7x10^{-6}	2.1x10^{-6}
PM	disease inc.	1.7x10^{-6}	1.7x10^{-6}	1.4x10^{-6}	1.3x10-3
IR	kBq U-235 eq	12.7	12.7	9.4	10.3
POF	kg NMVOC eq	0.20	0.19	0.20	0.16
AC	mol H+ eq	0.22	0.22	0.18	0.19
FE	kg P eq	0.02	0.02	0.02	0.20
ME	kg N eq	0.06	0.05	0.04	0.06
TE	mol N eq	0.40	0.40	0.30	0.40
RES-f	MJ	1543	1526	1112	1186
RES-m	kg Sb eq	1.0x10^{-4}	1.0x10^{-4}	1.3x10^{-4}	1.4x10^{-4}
WAT	m3 depriv.	19.4	19.1	18.5	14.2
LU	Pt	111	110	97	109
HT-nc	CTUh	2.7x10^{-7}	2.7x10^{-7}	3.3x10^{-7}0	2.7x10^{-7}
HT-c	CTUh	1.7x10^{-7}	1.6x10^{-7}	1.3x10^{-7}	1.74x10^{-7}
FWT	CTUe	253	224	330	330

Flax fiber reinforced composites

Table S20: LCA results flax fiber reinforced composite

Indicator	Unit	Reference	Landfill	Incineration	Chemical	Mechanical
CC	kg CO2 eq	9.4	8.0	12.0	26.0	7.2
ODP	kg CFC11 eq	3.1x10^{-7}	2.7x10^{-7}	3.1x10^{-7}	4.8x10^{-7}	2.3x10^{-7}
PM	disease inc.	4.1x10^{-7}	3.5x10^{-7}	4.0x10^{-7}	7.0x10^{-7}	2.9x10^{-7}
IR	kBq U-235 eq	1.09	1.12	1.22	2.85	1.53
POF	kg NMVOC eq	0.40	0.03	0.04	0.06	0.03
AC	mol H+ eq	0.60	0.05	0.06	0.06	0.05
FE	kg P eq	0.003	0.003	0.004	0.008	0.003
ME	kg N eq	0.04	0.03	0.03	0.03	0.02
TE	mol N eq	0.11	0.10	0.11	0.16	0.09
RES-f	MJ	164	141	160	319	132
RES-m	kg Sb eq	6.2x10^{-5}	5.8x10^{-5}	6.4x10^{-5}	11.9x10^{-5}	5.9x10^{-5}
WAT	m3 depriv.	3.10	2.80	3.19	9.50	2.50
LU	Pt	378	376	378	256	293
HT-nc	CTUh	1.48x10^{-7}	1.45x10^{-7}	1.57x10^{-7}	2.7x10^{-7}	1.28x10^{-7}
HT-c	CTUh	5.3x10^{-8}	4.07x10^{-8}	4.8x10^{-8}	6.6x10^{-7}	3.4x10^{-7}
FWT	CTUe	191	152	213	282	120

Discussions about improvements

Bisphenol A and resorcinol

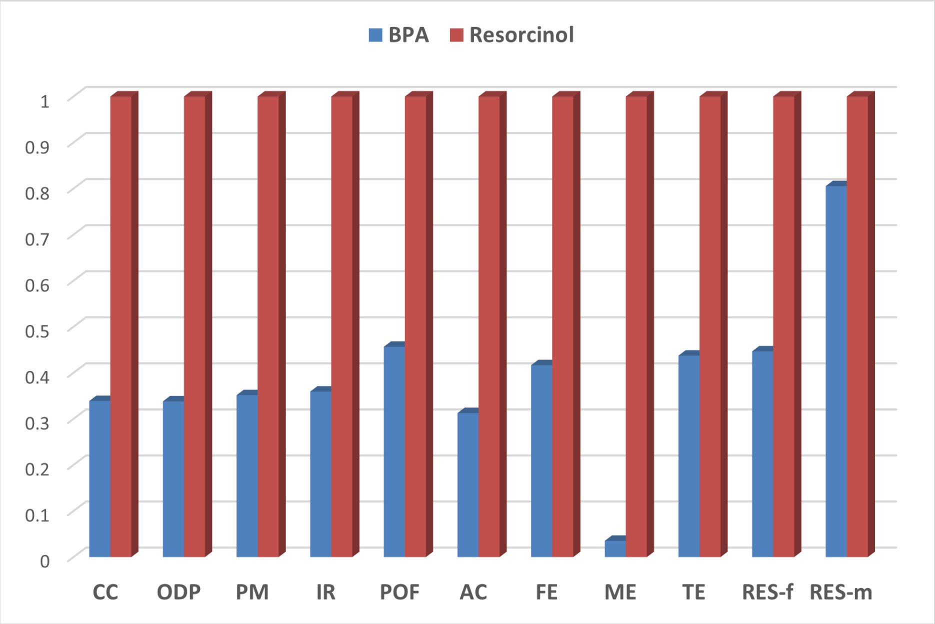

Figure S5: Comparative EI between bisphenol-A (BPA) and resorcinol for 1 kg, obtained from EcoInvent 3.4 database.

Bio-epichlorohydrin interest

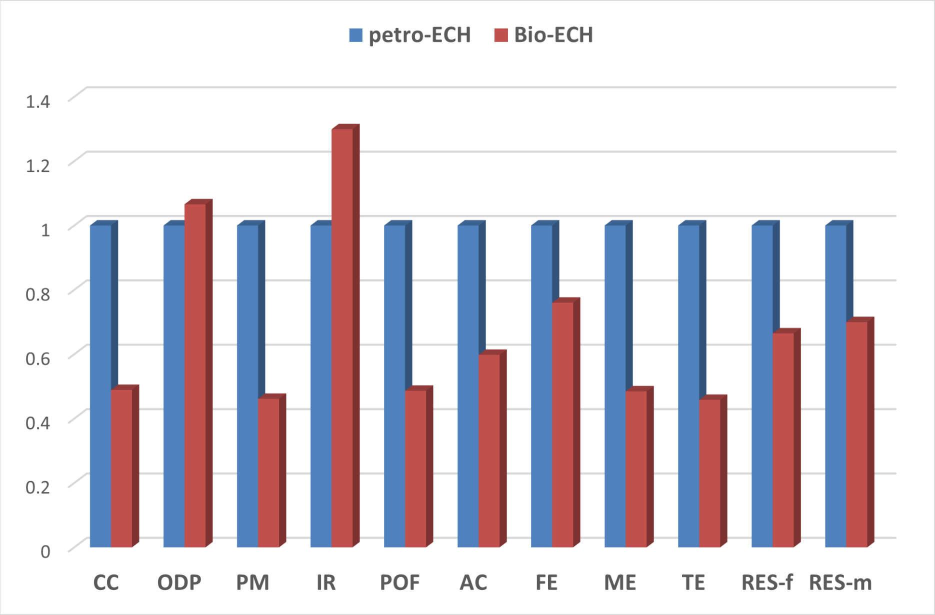

Figure S6: Comparison of EI of biobased (glycerol) and petro-based (allyl chloride) epichlorohydrin.

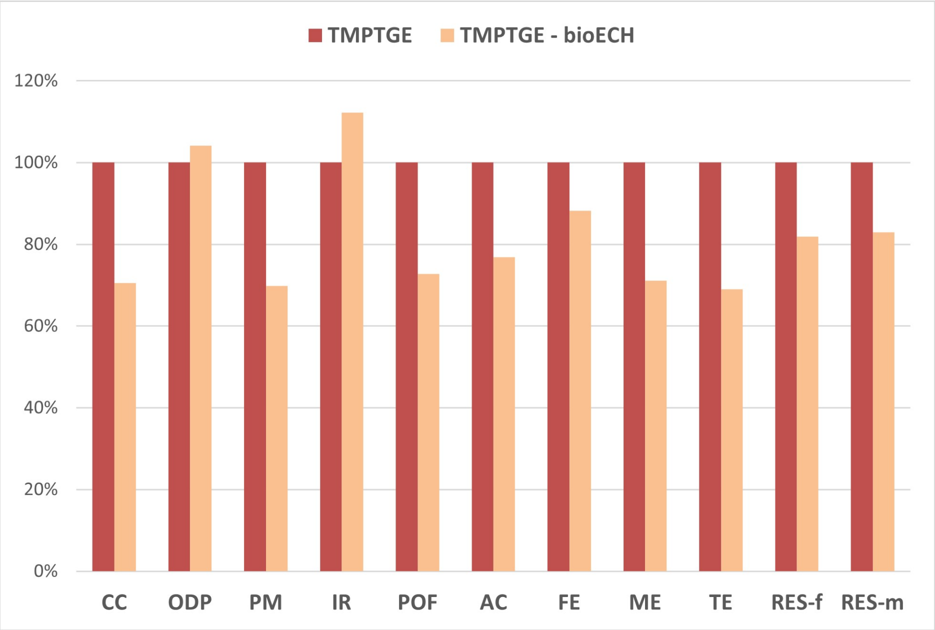

Figure S7: Comparison of EI of TMPTGE from petro-based and biobased epichlorohydrin.

Impact of acidolysis and improvements

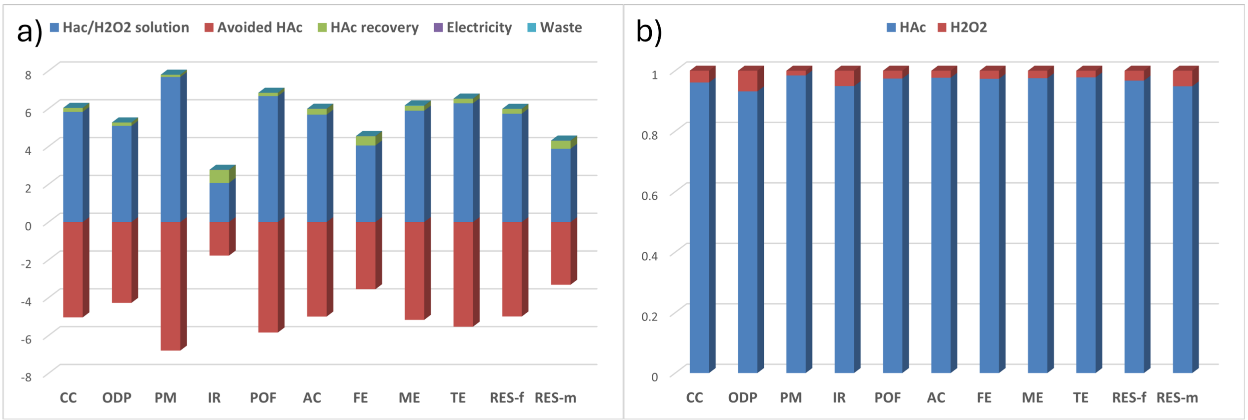

Figure S8: EI of the chemical recycling process and importance of HAc. a) contribution of each constituent in the recycling process, and b) shares of HAc in the depolymerization solution mixture.

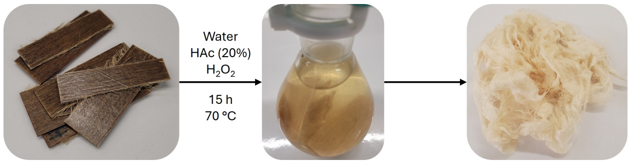

Figure S9: Pictures of the recycling of the flax/hybrid composite in 20% acetic acid.

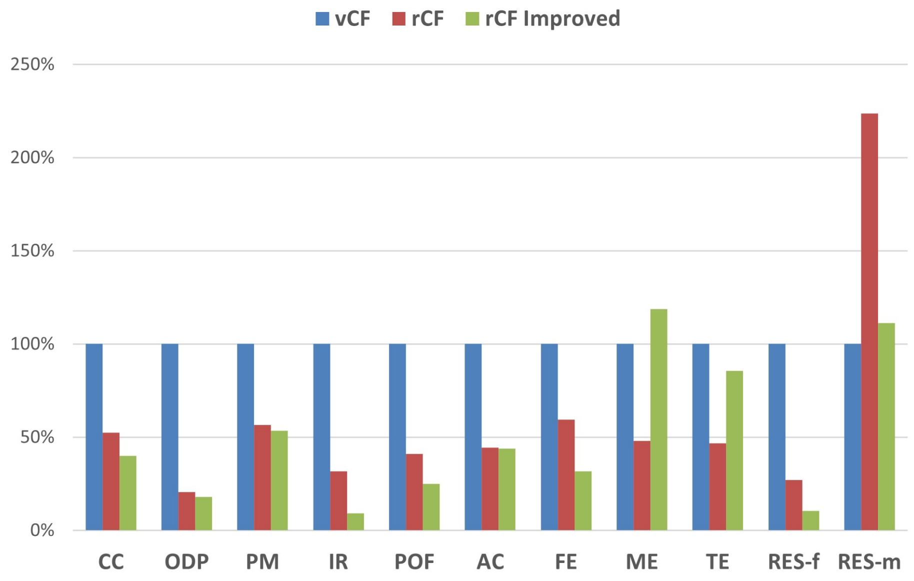

Figure S10: Improvement of the chemical recycling processes for carbon fiber reinforced composite. Comparison of the environmental footprint of virgin carbon fibers (vCF) vs the glacial acetic acid process (rCF) and the vinegar-based process.

References

1. Liang, C.; Jadidi, Y.; Chen, Y.; Gracida-Alvarez, U.; Torkelson, J. M.; Hawkins, T. R.; Dunn, J. B. Techno-economic Analysis and Life Cycle Assessment of Biomass-Derived Polyhydroxyurethane and Nonisocyanate Polythiourethane Production and Reprocessing. *ACS Sustainable Chemistry & Engineering* **2024**, *12*, 12161–12170, DOI: 10.1021/acssuschemeng.4c04046.

2. Jacquet, L.; Le Duigou, A.; Kerbrat, O. A Proposal for a Carbon Fibre-Manufacturing Life-Cycle Inventory: A Case Study from the Competitive Sailing Boat Industry. *Journal of Composites Science* **2024**, *8*, 276, DOI: 10.3390/jcs8070276.
